# *Bank1* deficiency reshapes the gut microbiota of lupus mice towards an anti-inflammatory composition

**DOI:** 10.3389/fimmu.2025.1586025

**Published:** 2025-07-21

**Authors:** Georgina Galicia, María Botía-Sánchez, Daniel Toro-Dominguez, Ana López García, Juan Rafael Valera, Gonzalo Gómez Hernández, Raquel Marcos Fernandez, Noelia Carmona, Gracia Luque, María Morell, Nieves Varela, Francisco Pérez-Cozar, Abelardo Margolles, Margarita Aguilera, Marta E. Alarcón-Riquelme

**Affiliations:** ^1^ Genetics and Genomics of Immune-Mediated Diseases, Centro Pfizer – Universidad de Granada – Junta de Andalucía de Genómica e Investigación Oncológica (GENYO), Granada, Spain; ^2^ Institute for Environmental Medicine, Karolinska Institute, Stockholm, Sweden; ^3^ Department of Microbiology and Biochemistry of Dairy Products, Instituto de Productos Lácteos de Asturias (IPLA), Consejo Superior de Investigaciones Científicas (CSIC), Villaviciosa, Spain; ^4^ Health Research Institute of Asturias (ISPA), Avenida Hospital Universitario s/n, Oviedo, Spain; ^5^ Institute of Nutrition and Food Technology “José Mataix”, Center of Biomedical Research, University of Granada, Granada, Spain

**Keywords:** microbiota, IgA, systemic lupus erythematosus (SLE), TLR7, BANK1, genetics

## Abstract

The B-cell scaffold protein with ankyrin repeats (BANK1) regulates Toll-like receptor-7 (TLR7) signaling in B cells and its absence ameliorates lupus. Here, we investigated the involvement of *Bank1* in the gut mucosal B cell response to commensals in a murine model of lupus. In health and disease, *Bank1* deficiency resulted in changes in the intestinal IgA production levels that showed differential bacterial binding associated with a re-organization on the composition and structure of the gut microbiota. Furthermore, the amelioration of lupus gut pathology in mice lacking *Bank1* was linked to the increase of *Parabacteroides distasonis* that when vertically transmitted or orally administered, as emerging probiotic, reduced disease severity and repaired signs of distorted intestinal permeability. The increase of *P. distasonis* directly correlated with anti-inflammatory processes. *In vitro* stimulation either with *P. distasonis* or via TLR9 allowed for the differentiation of IL-10 producing B cells which, *in vivo*, differentially accumulated in the Peyer´s patches of *Bank1*-deficient lupus mice. Finally, the blood microbiome of lupus patients was found to be devoid of *P. distasonis*, whereas healthy controls exhibited the bacterium, thereby supporting the protective role of *P. distasonis* in the disease.

## Introduction

Lupus is a systemic and complex autoimmune disease with multiorgan involvement and multifactorial etiology. The loss of tolerance in lupus relies on the interplay of genetic and environmental factors (1). Among the genetic variants, certain alleles regulating the B cell activation pathway related with Toll-like receptor-7 (TLR7) have been associated with a higher risk of developing severe forms of lupus with early onset (2, 3).

The B-cell scaffold protein with ankyrin repeats 1 (*BANK1*), is a well-known susceptibility gene for lupus (4), particularly associated in African Americans (5, 6). *BANK1* is mainly expressed in mature B cells, specifically in activated memory B cells (7). It is also one of the earliest markers of pre-memory B cells within germinal centers (8). BANK1 protein contains a TIR domain and TRAF6-binding motifs allowing it to bind MyD88 and TRAF6, respectively, showing its involvement in TLR7 and 9 signaling (9). The human gene variants of *BANK1* show differential signaling upon CD40 and BCR activation of peripheral blood mononuclear cells (PBMC), all with altered expression of *FOXO1* and *AKT* (10). Moreover, *BANK1* RNA levels have also been reported to be increased in blood B cells from kidney-transplanted tolerant patients (11), but without modifying IL-10 nor IL-6 production in these B cells (11, 12). However, in the so-called regulatory B cells (Breg), including CD24^+^CD38^+^, IL-10^+^ and Granzyme B^+^ B cells, *BANK1* expression was shown to be significantly downregulated (12, 13), indicating divergent roles of BANK1 in effector and regulatory B cell subsets.

Regulatory B cells are known for being key mediators in restraining the immune response and maintaining homeostasis. In lupus patients, IL-10-producing regulatory B cells have been shown to be significantly impaired in number and function, with memory IL-10^+^ B cells showing the most marked reduction compared with healthy controls (14). Additionally, in lupus nephritis patients it has been shown both decreased production of IL-10 and inability of IL-10^+^ B cells to suppress CD4^+^ T cell cytokine production (15). Furthermore, Breg cells have been demonstrated to dampen inflammation in murine models of lupus (16, 17). However, their role in lupus pathogenesis and their association with genetic susceptibility variants remain unexplored.

Implications of *Bank1* deficiency have also been proved in murine models of lupus. In mice, *Bank1* is expressed in mature B cells, and mice knockout for *Bank1* (*Bank1^-/-^*) present with normal development of immune cell subsets. However, *Bank1* ^-/-^ mice show increased formation of germinal centers, T cell-dependent IgM responses, B cell proliferation and survival along with augmented Akt activation upon CD40/CD40L binding compared with *Bank1* sufficient mice (18). Similarly, stimulation with CD40L combined with CpG, increased Akt-phosphorylation in splenic *Bank1^-/-^* B cells (19), suggesting that *Bank*1 signaling participates in the T cell-B cell interaction. The absence of *Bank1* in the lupus B6.*Sle1*.*yaa* mice resulted in a delay in disease development and progression. The mice presented a decreased production of IgG anti-dsDNA antibodies, reduced type I interferon signaling and *Stat1* activation, diminished expression of *Ifnb, Ifna4, Irf7*, and *Aicda* in B cells, and ameliorated glomerulonephritis, all while restoring the normal percentages of B220^+^IgM^+^IgD^hi^ B cells (20). Furthermore, deficiency of *Bank1* in TLR7-transgenic (TLR7Tg) mice leads to a reduction in age-associated B cells (ABCs) and in an IFN-stimulated gene expressing B cell population, otherwise, increased in the TLR7Tg.*Bank1^+/+^* lupus mice. In addition, the absence of *Bank1* modifies the gene expression pattern of the ABCs being pro-inflammatory to anti-inflammatory (21).

During the recent decade, gut microbiota has emerged as an increasingly important environmental factor in lupus pathogenesis. Gut bacteria are known for shaping the immune response, while the gut mucosal B cell response is critical in maintaining a diverse and balanced microbiota. This interplay is mainly mediated by the production of secretory IgA that binds commensals, shaping microbiota composition (22), which in turn modulates the maturation of the immune system and eventually the systemic immune response (23). In lupus, gut dysbiosis or altered microbiota have been pointed out as an environmental factor with a causal relationship between the gut microbiome composition and the risk of developing disease (24). The gut microbiome of lupus patients showed a consistent reduction in the Bacillota/Bacteroidota (formerly known as Firmicutes/Bacteroidetes) ratio and taxa diversity compared with healthy controls regardless of their lifestyle, geographical location, or disease stage (25).

One mechanism suggested for microbiota to trigger the autoimmune response is via antigen mimicry, as the orthologs of human autoantigens Ro60 and Sm and the mimotope β2-glycoprotein I (β2GPI) have been found to be expressed in mucosal commensals (26). Furthermore, gut microbiota abundance alterations, such as *Lactobacillus reuteri* or *Bacteroides acidifaciens* have been linked to increased gut epithelial barrier permeability, which allowed the translocation of intestinal bacteria that triggered the activation of T and B cells and plasmacytoid dendritic cells (pDC), contributing to lupus progression (27, 28). Additionally, the genetic background of the host is a factor contributing to the establishment and composition of the microbiota, as several genetic variants have been associated with the abundance of specific bacterial species in humans and mice (29).

In this work, we aimed at understanding how the interaction of host genetics and gut microbiota determine lupus disease outcome. Although previous observations of dysregulated TLR7, TLR9, and MyD88 signaling in splenic *Bank1^-/-^* B cells anticipate that *Bank1* may drive the mucosal B cell response, the role of *Bank1* in the gut mucosal B cell response has not yet been addressed. Here, we report that absence of *Bank1* resulted in altered intestinal IgA production that was associated with changes in the intestinal microbiota composition and improved disease outcomes. The reduced lupus inflammatory response was directly correlated with an increase of *Parabacteroides distasonis*, a reduction of intestinal permeability, and the induction of IL-10-producing B cells.

## Methods

### Mouse strains and husbandry

C57Bl/6 wild-type (WT) mice were purchased from Charles River and *Bank1^-/-^* mice in C57Bl/6 background were a gift from Dr. T. Kurosaki (RIKEN Institute, Kyoto, Japan). Transgenic mice carrying 8–16 copies of the *Tlr7* gene in the Y chromosome (TLR7Tg,6) were maintained on a C57Bl/6J genetic background. TLR7Tg.6 mouse strain here named TLR7Tg was obtained from Dr. Darise Farris, Oklahoma Medical Research Foundation, United States (30). *Bank1^-/-^* mice were crossed with *Tlr7*Tg mice to obtain TLR7Tg *Bank1^-/-^* mice. Animals were housed and bred under pathogen-free (SPF) conditions in the animal facility of the Biomedical Research Centre of Granada University. All mice were kept in cages with filtered air that were refreshed once a week by trained personnel. Animals were fed with commercial standard chow and sterile water *at libitum.* Mice were maintained at standard conditions of daylight (12 hours light-dark cycle), humidity, and temperature.

In the TLR7Tg model, male mice developed lupus in a progressive and spontaneous manner. The lupus-like signs and symptoms were unambiguously identified in male mice from week 23 to week 32 (endpoint) (30). As the spontaneous model developed in male mice for the induced model, imiquimod (IMQ)-induced lupus was triggered in 12-week-old male mice by topical application of the TLR7 agonist IMQ (1.5 mg of 5% Aldara) on the left ear every other day for 8 weeks. Although this lupus model was developed in male mice, these results can be extrapolated to females as the increased *TLR7* gene dosage also results in lupus development in female mice (27).

### Littermate experiments

According to Robertson et al. (31), one male WT and two female *Bank1* ^-/-^ mice were bred to obtain *Bank1^+/-^* (F1) mice. Two females and one male F1 were bred to obtain an F2 littermate generation. The three-week-old F2 littermates were genotyped and weaned and were then evenly distributed across cages according to their genotype *Bank1 ^+/+^*, *Bank1^-/+^*, and *Bank1 ^-/-^*.

### Blood and fecal sample collection

Mice were anesthetized with ketamine at 100mg/kg and xylazine at 5mg/kg in PBS to collect blood through exsanguination via cardiac puncture. In addition, blood samples were collected from the saphenous vein at various times during the disease course with Microvette^®^ capillaries (Sarstedt). Blood was allowed to clot overnight at 4°C and then centrifuged at 9560 g for 10 minutes to obtain serum. Serum samples were stored at -20°C until use. Fecal samples were collected under sterile conditions at various times during the disease course. Feces were immediately stored at -20°C until processed.

### Bacterial isolation from feces

Fecal matter was dissolved in sterile PBS (GIBCO) at 100 mg/ml. To remove undigested food, the fecal slurry was centrifuged at 187 g for 10 minutes. The supernatant, containing bacteria, was then centrifuged at 2020 g for 10 minutes. For the detection of fecal IgA, the supernatant was collected and mixed with a protease inhibitor cocktail (Roche) and stored at -20°C until analysis. The bacterial pellet was washed twice with PBS 1% BSA for direct analysis.

### *Ex vivo* characterization of IgA binding to fecal bacteria

Bacteria isolated from fecal content were resuspended in 100 μl and transferred to a 96-well plate, where they were centrifuged at 2020 g for 5 minutes at 4°C. The bacterial pellet was resuspended and blocked with 1x BSA/1% PBS/20% rat serum. Blocked samples were washed with 1x BSA/1% PBS to be then incubated with anti-IgA APC antibody (eBioscience) diluted 1:40 in 1x/BSA 1% PBS. Bacteria were washed again and stained with 200 μl of SytoBC diluted 1:1000 in PBS 1x BSA/1%. Samples were then fixed in 2% PFA and then washed a final time to be resuspended in 200 μl for acquisition. All incubations in this protocol were performed for 15 min at 4°C in the dark.

### ELISA for the detection of immunoglobulins and autoantibodies

To assess anti-dsDNA antibody titers, 96-well ELISA plates (ThermoFisher Scientific) were coated with 100μl of protamine sulfate (Sigma) at 500 µg/ml for 45 minutes at 4°C, then 75μl of calf thymus dsDNA was added (Sigma Aldrich) at 5 µg/ml and the plates were incubated at 37°C for 2 hours followed by overnight incubation at 4°C. Plates were washed 5X with PBS containing 0,05% Tween 20 (PBST). Serum samples were added at dilutions from 1:200 to 1:2000 for the quantification of IgG and IgG2c. For IgA detection, serum samples were added at a 1:200 dilution in PBST/BSA 1%. Samples were incubated at 37°C for 2 hours. After two washes, either HRP-anti-IgG (1:1000 in PBST/BSA 1%, Southern Biotech), biotinylated anti-IgG2c (1:1000 in PBST/BSA 1%, Southern Biotech) or biotinylated anti-IgA (1:1000 in PBST/BSA 1%, Southern Biotech) was added and incubated at 37°C for 30 minutes. After 4X washing, HRP-streptavidin (1:100 in PBST/BSA 1%) was added and incubated for 30 minutes at room temperature. The plates were then washed 4X, 3,3’,5,5’-tetramethylbenzidine (TMB) substrate was added, and the colorimetric reaction was stopped by adding 2N H_2_SO_4_ (Sigma) after 10 minutes. Optical density (OD) was measured at 450 nm and 570 nm using an Infinite200Pro plate reader.

Total IgA in feces was measured by sandwich ELISA according to the manufacturer’s instructions (Invitrogen). Briefly, ELISA plates were coated with capture antibodies overnight at 4°C. After washing and blocking, samples diluted 1:50 were added and incubated for 2 hours at room temperature. The plates were then washed and HRP-conjugated anti-IgA detection antibody was added and incubated for 30 minutes. After washing 4X, TMB substrate was added. The reaction was stopped with 2N H_2_SO_4_ (Sigma). Absorbance was measured as described above.

### Flow cytometry

Peyer’s patches (PPs) were excised from the small intestine to be separately analyzed from the small intestine lamina propria (SILP) B cells. The small intestine was emptied, opened longitudinally, washed with cold HBSS, and cut into 0.5 cm pieces. Epithelial cells were removed by incubating the minced tissue in HBSS with EDTA 5mM 3X for 10 minutes at 37°C. The remaining tissue was digested with collagenase D (0.25 mg/ml) and DNAse (0.05 mg/ml, Roche) at 37°C and agitated at 250 rpm for 30 minutes to obtain a single-cell suspension, which was subsequently passed through 100 and 40 µm filters (BD). Peyer’s patches were also digested with collagenase D and DNase and passed through a 40 µm filter (BD) to obtain a single-cell suspension. The isolated cells were stained with live/dead aqua and then incubated with anti-CD16/32. This was followed by incubation with anti-CD19 FITC or BV605, -B220 eF450, PETxRed or FITC, -CD45 APC, -TCR PerCP Cy5.5, -GL7 PE, -IgD PE Cy7, -CD3 PE Cy7, -F4/80 PE Cy7, and -CD138 PercpCy5.5 (Biolegend). Cells were permeabilized (Cytofix/Cytoperm BD) for intracellular staining with anti-IgA PE (Southern Biotec), -IL-10 PE Cy7 (Biolegend). All antibodies were diluted 1:200 in PBS 1x/EDTA 2 mM/BSA 0.5% and incubated for 30 minutes at 4°C in dark. More in-depth information about all the antibodies included in this study is displayed in **Table 1**.

**Table 1:** Commercial details of the antibodies used for flow cytometry.

Target	Fluorochrome	Clone	Brand
CD19	FITC/BV605	6D5	Biolegend
B220	eF450/PE-TxRed/FITC	RA3-6B2	Biolegend
CD45	APC	30-F11	Biolegend
TCR	PerCP Cy5.5	H57-597	Biolegend
GL7	PE	GL-7	Biolegend
CD95	PE-CF594	JO2	Biolegend
IgD	PE Cy7	11-26c	Biolegend
CD3	PECy7	17 ^a^ 2	Biolegend
F4/80	PE Cy7	BM8	Biolegend
CD138	PerCP Cy5.5	28 1-2	Biolegend
IL-10	PE Cy7	JES5-16E3	Biolegend
IgA	PE	F1613-XN79B	Southern Biotech

### Immunofluorescence

The small intestine was dissected, and 2 cm segments of distal ileum were obtained, emptied, and opened longitudinally. Ileal segments were fixed in 4% PFA for 30 seconds, then washed in PBS, embedded in OCT (Tissue-Tek), and frozen in an isopentane-dry ice mix. Eight-µm sections were hydrated for 20 minutes in TBS and 20 minutes in TBS with Tween20 0.05% (TBS-T). Tissue sections were then blocked with TBS-T/BSA 5%, 2mg/ml of anti-CD16/CD32, and 10% normal rat and mouse serum for 30 minutes at room temperature. Slides were briefly washed in TBS-T and incubated with anti-EpCam APC at a 1:500 dilution (Invitrogen) and anti-Claudin 1 Alexa Fluor 488 at 1:50 dilution (Invitrogen) overnight at 4°C. Slides were briefly washed in TBS-T and cell nuclei were stained with Hoechst 33342 (1µM, Sigma-Aldrich) for 5 minutes at room temperature. Slides were mounted with SlowFade Diamond Antifade Medium (Thermo Fisher Scientific) and images of tissue sections were captured using a Zeiss 710 Laser Scanning Microscope, a Zeiss Plan-Apochromat 63X/1.40 NA oil-immersion DIC M27 objective (aperture pinhole= 1.0 Airy Unit), a Zeiss Plan-Apochromat 20X/0.8 NA objective and the Zeiss ZEN 2010 software. Fluorescence was acquired sequentially using different lasers for excitation and different photomultipliers for the detection of all fluorescence signals.

### Renal histology

Upon sacrifice, the right kidney was extracted, fixed in PFA 4%, and kept at 4°C for at least 48 hours. Kidneys were then incubated for 1 hour in formaldehyde followed by 45 minutes in 80% ethanol. Subsequent one hour incubations at increasing concentrations of ethanol solutions were done until reaching absolute ethanol. Lastly, the tissue was incubated for 1 hour in xylene before proceeding to its inclusion in paraffin. Paraffin-embedded formalin-fixed renal tissue was sectioned into 3μm sections and stained with periodic acid-Schiff (PAS) reagents for morphologic evaluation. To do this, the slides were incubated for 5 minutes in xylene, 5 minutes in absolute ethanol, 5 minutes in 70% ethanol, and 5 minutes in distilled water. Slides were then incubated for 25 minutes at room temperature in 0.5% periodic acid, followed by one washing with distilled water. Slides were then incubated for 40 minutes at room temperature with Schiff reactive, followed by a 5-minute wash with tap water. The final contrast was done with a 5 second incubation with hematoxylin.

To score renal damage, 10X optic micrographs were evaluated and characterized for a list of predefined alterations, using a semiquantitative scoring of the magnitude of the lesion. Briefly, images presenting mesangial or endocapillary hypercellularity were evaluated as grade 1; images with crescents and/or wire-loops in less than 50% of analyzed glomeruli were evaluated as grade 2; images with crescents and/or wire-loops in 50% or more of the analyzed glomeruli and images with alterations in capillary loops in less than 50% of analyzed glomeruli were classified as grade 3; images with alterations in capillary loops in more than 50% of analyzed glomeruli and without scarring or remarkable tubular damage were evaluated as grade 4; images with scarring, necrosis and/or remarkable tubular damage were classified as grade 5. Images without any of the previous alterations were evaluated as grade 0.

### *In vivo* gut permeability assay

Gut permeability *in vivo* was assessed by modifying the protocol described previously (27). Briefly, mice were weighted and fasted (food and water) for 4 hours, after which the mice were gavaged with fluorescein isothiocyanate (FITC)-coupled 4KDa dextran (Sigma) at 250 mg/kg dissolved in 1x PBS. Water was restored for 3 hours, and blood collected from the saphenous vein. Fluorescence was then measured in serum samples diluted 1:4 in 1x PBS using a plate reader (Infinite 200Pro). The excitation and emission wavelengths were set at 485 and 528 nm, respectively. The autofluorescence emission value of serum from untreated mice was subtracted from the experimental samples.

### B cell isolation and *in vitro* culture

B cells from single-cell suspensions of spleen, mesenteric lymph nodes, and PPs from 20-week-old mice, were isolated by negative selection with the EasySep Mouse B cell isolation kit (Stemcell) according to the manufacturer’s protocol. Isolated B cells were collected in RPMI media containing penicillin-streptomycin, non-essential amino acids, and beta-mercaptoethanol. For coculture with *P. distasonis*, 10^5^ B cells were plated in a 96 well-plate in contact with bacteria in a ratio of 10 cells to 1 bacterium for 48 hours. Four hours before harvesting, PMA, ionomycin, and monensin were added to determine IL-10 production. Alternatively, enriched B cells were stimulated either with anti-IgM (5μg/ml, Biolegend), anti-CD40 (5μg/ml, Biolegend), TGFβ (5ng/ml, R&D Systems), and retinoic acid (1μM, Sigma). To assess IgA class switching, cells were cultured for 96 hours and harvested to be stained with live/dead far red and then incubated with anti-CD16/32. This was followed by incubation with anti-CD19 BV605, -B220 PECy7, -IgD APCCy7, -CD138 BV500, -α4β7 PE (Biolegend). Cells were then permeabilized (Cytofix/Cytoperm BD) for intracellular staining with anti-IgA FITC (Southern Biotec). All antibodies were diluted 1:200 in PBS 1x/EDTA 2 mM/BSA 0.5%.

### DNA extraction

Fecal microbial DNA was extracted from fecal samples using the QIAamp^®^ Fast DNA Stool Mini Kit “Pathogen Detection” (QIAcube/QIAGEN). Blood microbial DNA was extracted from 200 μl of plasma samples from the set of lupus patients from the PRECISESADS project (32) using the Norgen Biotek Corp columns for circulating DNA purification kit micro (SKU 55500). Both kitoma samples and blood withdrawal skin-puncture-site DNA controls were also obtained with the same Norgen Biotek columns. Quality and quantity were assessed by NanoDrop (ThermoFisher).

### 16S rRNA gene sequencing

Amplicon libraries for the 16S rRNA gene hypervariable region (V4) were generated using 515F–806R primers (Forward 5’-GTGYCAGCMGCCGCGGTAA-3’ and Reverse 5’-GGACTACNVGGGTWTCTAAT-3’). The resulting amplifications were analyzed by electrophoresis in a 2% agarose gel. Amplified products were then quantified by Qubit (ThermoFisher), pooled using 50ng/sample, and filtered in a column (Omega Bio-Tek) using DNA AMPure XP magnetic beads (Beckman Coulter) to avoid non-genetic waste from contaminating the sequencing step. Quality control was performed by High Sensitivity Bioanalyzer (Agilent) to check that there were no remaining primers. The size of the library was the expected 400bp. The resulting purified pool was diluted to 4nM, quantified again by Qubit, and sequenced using the MiSeq Reagent Kit v3 in a MiSeq System according to Illumina protocols.

### 16S data preprocessing, OTU assignment, and diversity analyses

The entire pipeline was carried out using R and Python programming languages and QIIME2 software. First, quality control was performed from raw sequencing reads using *q2-dada2* plugin from QIIME2, setting a Q20 as minimum Phred Score. The end-50 pairs of bases of reads were removed. Afterwards, operational taxonomic units (OTUs) were identified using the *q2-vsearch* plugin from QIIME2, grouping the sequences with an identity above 99%. OTUs from environmental microorganism contamination during sample handling were identified and removed using *decontam* R package and either negative control for fecal samples or kitoma and skin puncture site for plasma samples, were used as reference. For OTUs to taxa annotation, a sklearn-based taxonomic classifier was built using the *q2-feature-classifier* plugin from QIIME2 software, employing the reference sequences of 16S rRNA genes available in the GreenGenes v.13.8 database. Unaligned sequences were filtered, and a maximum likelihood-based phylogenetic tree was constructed using the MAFFT and RAxML algorithm from *q2-alignment* and *q2-phylogeny* plugin from QIIME2, respectively.

Diversity analysis between groups of samples was performed using the taxa counts matrix and the phylogenetic tree. Alpha diversity was evaluated by the Kruskal-Wallis test obtaining Faith’s Phylogenetic Diversity (FPD), Pielou’s Evenness and Shannon Index values. Beta diversity was estimated using Bray-Curtis dissimilarity, Jaccard Index and Weighted and Unweighted Uniformal Fraction (UniFrac) distance using permutational multivariate analysis of variance (PERMANOVA) test. Finally, LEfSe (linear discriminant analysis effect size) was performed to measure the effect size of each taxon for each group of samples and the significance level was assessed by the Kruskal-Wallis test.

To determine the most relevant differential species in the microbiome, 16S rRNA sequencing-derived taxonomic units were filtered by Wilcoxon rank-sum test to select the differentially significant taxa (p<0.05), then to Recursive Feature Elimination (RFE) with a 10-fold cross-validation (rfeControl() function.

### qPCR of *Parabacteroides distasonis* and *Bacteroides acidifaciens*

A standard curve was prepared from *Parabacteroides disatsoni*s and *Bacteroides acidifaciens* by microscopically counting the number of bacterial cells. Initial 10^8^ total bacteria were serially diluted up to 10^1^. The DNA from each dilution was extracted and purified and 5μl from each dilution was used for qPCR. From fecal samples’ DNA, 20ng were used for PCR amplification and Ct values from fecal samples were interpolated in the standard curve to estimate the number of bacteria in each sample. Primers used were *Parabacteroides distasonis* (Forward 5’-GGACACGTCCCGCACTTTAT-3’ Reverse 5’-TTCTGAGAGGAAGGTCCCCC-3’), *Bacteroides acidifaciens* (Forward 5’-GTATGGGATGGGGATGCGTT-3’ Reverse 5’-CTGCCTCCCGTAGAGTTTGG-3’). The PCR reactions were carried out in Sybrgreen mix (Thermo) in a final volume of 20μl (95.0°C 10 minutes 95.0°C15 sec 60.0°C 1 minutes).

### Bacterial strains and growth conditions

*Bacteroides acidifaciens* DSM15896 and *Parabacteroides distasonis* DSM20701 were obtained from the Leibniz Institute DSMZ-German Collection of Microorganisms and Cell Cultures GmbH. The culture medium for *Bacteroides acidifaciens* DSM15896 was 50% (v/v) of Brain-Heart Infusion (BHI, Oxoid Ltd) and 50% (v/v) of Reinforced Clostridial Medium (RCM, Oxoid Ltd)-supplemented with 5% (v/v) heat-inactivated fetal bovine serum (Sigma). *Parabacteroides distasonis* DSM20701 were cultured in Gifu Anaerobic Medium (GAM; HiMedia Laboratories). Both bacteria were first grown on the surface of agar plates at 37°C in an MG500 anaerobic chamber (Don Whitley Scientific; atmosphere of 10% (v/v) H_2_, 10% CO_2_, and 80% N_2_) for 48 hours. Subsequently, isolated colonies were inoculated in broth media and incubated O/N, and these pre-cultures were used as fresh inoculum for the preparation of 1000 ml broth cultures. After O/N growth, cells were washed with PBS and pellets were resuspended in 30ml of the corresponding medium supplemented with 20% trehalose. Bacterial stocks of 1ml were preserved at -80°C until use. The identity of the bacterial stocks was corroborated by partially sequencing the 16S rRNA gene using the primers (33, 34) 27-Forward 5’-AGAGTTTGATCCTGGCTCAG-3’ and 1492-Reverse 5’-GGTTACCTTGTTACGACTT-3’.

### Translocated bacterial growth

Mesenteric lymph nodes were collected in sterility and immediately frozen in liquid nitrogen. Mesenteric lymphoid tissue was thawed just before being homogenized with two glass slides in an anaerobic workstation (Whitley A45 HEPA) with atmosphere of 5% (v/v) H_2_, 5% CO_2_, and 90% N_2_ to then culture in 5ml of sheep blood-rumen Luria-Bertani anaerobic media. Culture was maintained in anaerobic conditions at 37°C for 72 hours. One ml sample was taken to extract the DNA of grown bacteria for 16S rRNA gene V4 amplicon sequencing.

### Oral administration of bacteria

Mice received 10^9^ bacterial cells of *Parabacteroides distasonis* in 200μl PBS daily by gavage for 2 weeks before and during the 8 weeks of IMQ treatment. Control mice received 200μl of sterile PBS daily. The presence of *Parabacteroides distasonis* in fecal samples was determined by qPCR.

### Semi-polar metabolite (reverse-phase) profiling

Cecal contents (50 mg) were placed into 2 mL reaction tubes. After the addition of extraction solvent (MQW), the samples were homogenized by bead-beating for 5 minutes. The tubes were then centrifuged at 16,000 g for 10 minutes at 4°C to separate the supernatant. To filter the samples, supernatants were transferred to SpinX centrifuge filters and centrifuged again at 15,000 g for 5 minutes at 4°C. The filtrates were collected for further analysis. To ensure high quality sample preparation, a quality control sample (QC sample) was prepared by pooling (60 µL) equal aliquots from each sample, to create a representative average of the entire set. This QC sample was processed and analyzed after every sixth experimental sample throughout the sample analysis sequence. Samples were diluted 11 times in mobile phase eluent A (10mM ammonium formate + 0.1% formic acid in ultrapure water) and spiked with stable isotope labelled standards before analysis. The samples were analyzed with MS-Omics’ semi-polar metabolites method, using a randomized sample order using a UPLC system (Vanquish, Thermo Fisher Scientific) coupled to a high-resolution quadrupole Orbitrap mass spectrometer (Orbitrap Exploris 240 MS, Thermo Fisher Scientific). Ionization was achieved using an electrospray ionization interface operated in positive and negative ionization mode under polarity switching.

Samples were analyzed by liquid chromatography with tandem mass spectrometry (LC-MS/MS) fragmentation mode for the identification of compounds. Annotations are based on three pieces of information: accurate mass, MS/MS spectra and known retention time obtained from standards analyzed on the same system. Annotations are reported at level 1, which is the most confident identification.

### Statistics

Data was analyzed using GraphPad Prism 9.2.0 and R. Non-parametric unpaired Mann-Whitney test was used to make the comparisons between groups unless stated differently. P ≤ 0.05 values were considered significant.

## Results

### The absence of *Bank1* modifies intestinal B cell populations and lupus-induced severity

**Figure 1:**
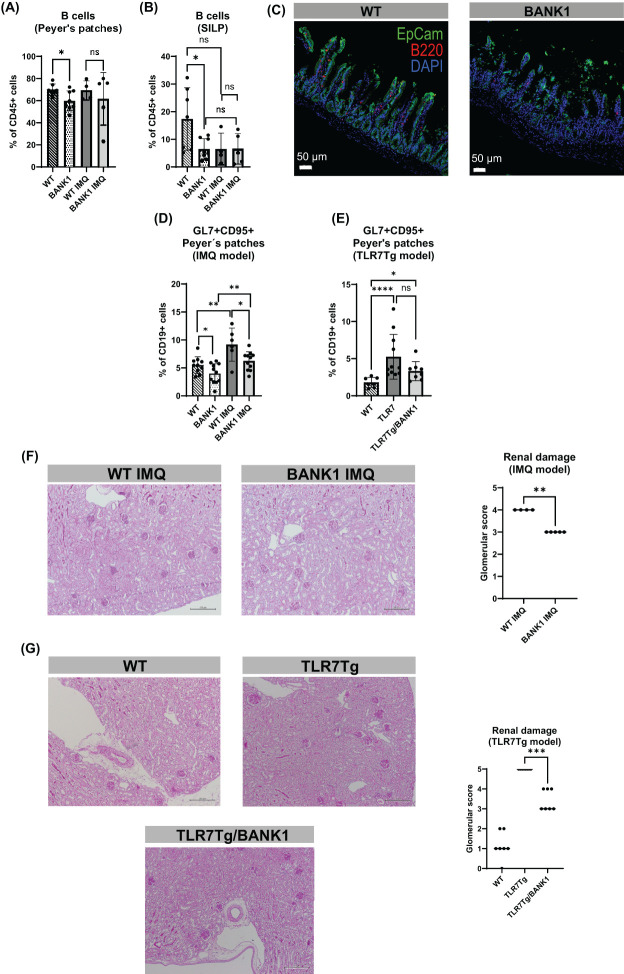
Bank1 deficiency downregulates the gut-associated inflammation of IMQ-induced and TLR7Tg-mediated lupus. **(A)** Flow cytometric analysis of B cells (gated as CD19^+^B220^+^ from CD45^+^CD11c^-^F4/80^-^ cell population) from PPs and **(B)** SILP. **(C)** Fluorescent staining of B cells in longitudinal sections of the ileum, Ep-CAM-aF488 (green), B220-PE (red), nuclei-DAPI (blue). **(D)** Frequency of GC B cells (gated as GL7^+^CD95^+^ cells from CD45^+^CD19^+^ cell population) from IMQ model and **(E)** TLR7Tg model. **(F)** PAS staining of longitudinal sections of the kidneys and glomerular score for the **(F)** IMQ model and **(G)** TLR7Tg model. Statistical analysis performed by Mann-Whitney unpaired test. Data represent the results of 3–5 independent experiments with similar results and n=5–11 mice per group. BANK1 = *Bank1* ^-/-^ mice, TLR7Tg/BANK1 = TLR7Tg.*Bank1* ^-/-^. ns (non-significant): ***p ≤ 0.001.

To study the role of *Bank1* in the intestinal lupus pathology, we used two TLR7-dependent lupus models, the inducible (IMQ-mediated) and the spontaneous TLR7 transgenic lupus models. Because of the involvement of BANK1 in B cell signaling, we first assessed the gut-associated lymphoid tissue (GALT)-resident B cell subpopulations. The Peyer’s patches (PPs) B cells frequency of naive *Bank1^-/-^* mice was reduced compared to naive WT mice, whereas PPs of IMQ-treated *Bank1^-/-^* and IMQ-treated WT mice showed comparable B cell frequencies (**Figure 1A**). Similarly, in the small intestine lamina propria (SILP) of naïve *Bank1^-/-^* mice there was a significant reduction in the frequency of B cells compared with their naïve WT counterparts (**Figures 1B, C**). Additionally, in both IMQ-treated *Bank1^-/-^* and IMQ-treated WT mice, the B cell frequency was reduced in the SILP in comparison to naïve WT mice (**Figure 1B**). In the mesenteric lymph nodes (mLN) the B cell frequency was similar between *Bank1^-/-^* and WT naïve mice. However, upon IMQ-treatment, the frequency of B cells was higher in *Bank1^-/-^* mice than in WT mice (**Supplementary Figure S1A**).

The evaluation of germinal center (GC) formation revealed that the absence of *Bank1* resulted in a lower frequency of germinal center (GC) B cells in the PPs of naïve *Bank1^-/-^* mice in comparison to naïve WT mice (**Figure 1D**; **Supplementary Figure S1B**). However, following IMQ treatment, the frequency of GC B cells in the PPs was increased in both genotypes compared with their naïve counterparts. Nevertheless, this increase was less pronounced in IMQ-treated *Bank1^-/-^* mice, which exhibited a lower frequency of GC B cells than IMQ-treated WT mice (**Figure 1D**). Likewise, in the TLR7Tg model, the frequency of GC B cells in the PPs was elevated in TLR7Tg and TLR7Tg.*Bank1^-/-^* mice relative to WT naïve mice. However, the frequency of GC B cells was diminished in TLR7Tg.*Bank1^-/-^* mice, although not significantly, in comparison to TLR7Tg mice (**Figure 1E**). The increased GC activity observed in the PPs mirrored the GC activity in the spleen of lupus mice (**Supplementary Figure S1C**).

Concomitant with the reduction in gut inflammation in *Bank1* deficient mice, we observed decreased splenomegaly (**Supplementary Figure S1D**) and lower serum IgG2c anti-dsDNA antibody levels compared with IMQ-treated WT and TLR7Tg mice (**Supplementary Figure S1E**). The evaluation of renal damage revealed that IMQ-treated *Bank1^-/-^* lupus mice experienced a reduction in glomerulonephritis compared with IMQ-treated WT controls (**Figure 1F**). Similarly, TLR7Tg.*Bank1^-/-^* mice displayed a reduction in renal damage in comparison to TLR7Tg controls (**Figure 1G**). Some images of the most representative lesions found in lupus mice are depicted in **Supplementary Figure S1F**. Altogether, the above exposed results indicate that *Bank1* ^-/-^ present milder manifestations of lupus.

### *Bank1* modulates lupus-associated intestinal permeability and commensal translocation

**Figure 2:**
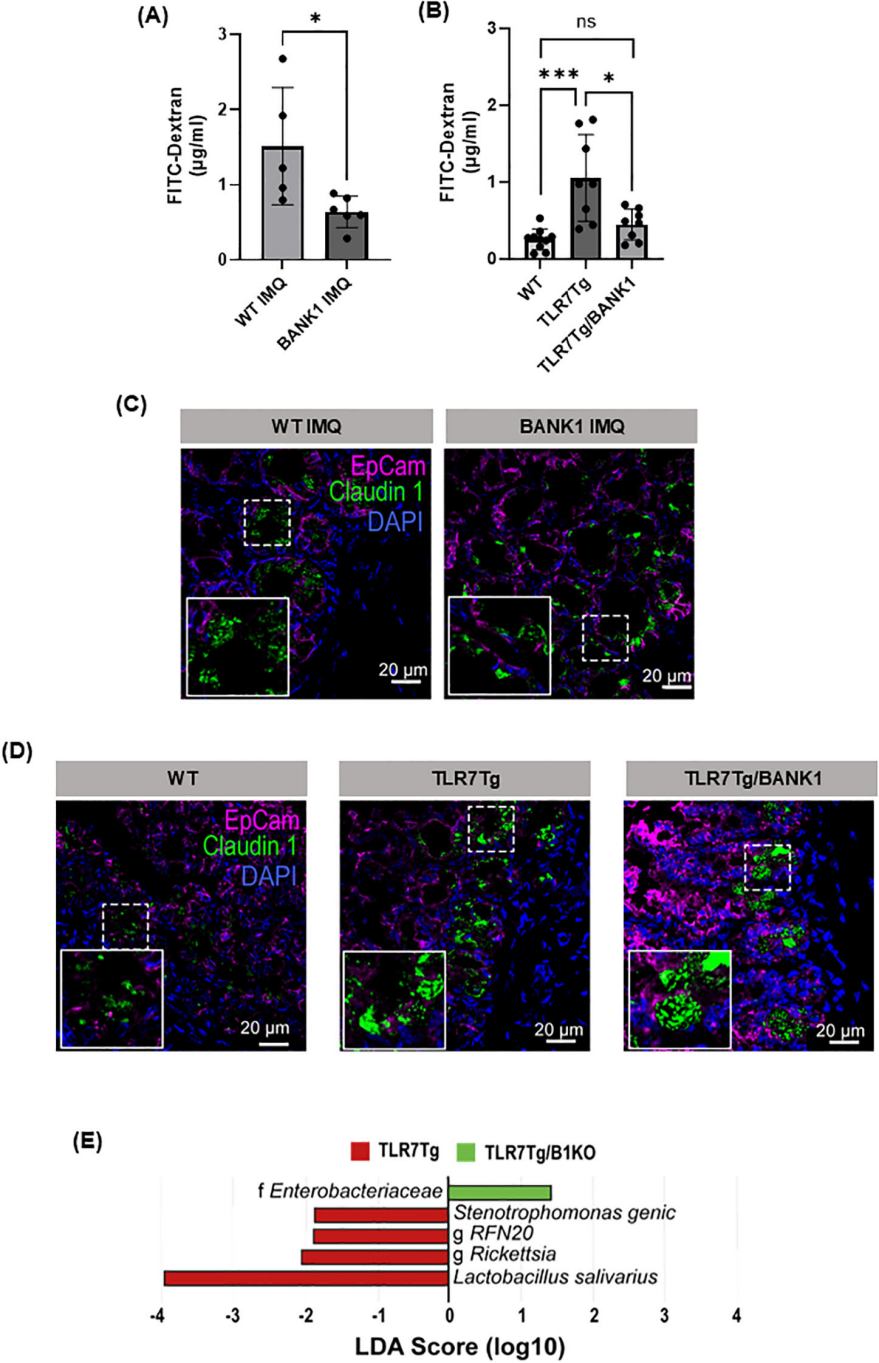
Bank1 regulates the increased intestinal permeability that results of TLR7 activation. **(A)** Gut permeability assessment *in vivo* with FITC-dextran in IMQ-induced lupus WT and *Bank1^-/-^* mice and **(B)** TLR7Tg lupus model in *Bank1^+/+^* and *Bank1^-/-^* mice. Representative fluorescent staining of Claudin-1 expression in transversal sections of the distal ileum of **(C)** IMQ-treated mice and **(D)** TLR7Tg mice. Claudin 1- aF488 (green), EpCam-APC (pink), nuclei-DAPI (blue). **(E)** LEfSe plot of translocated taxa found in mesenteric lymph nodes. Statistical analysis performed by Mann-Whitney unpaired test. Data represents results from 3 independent experiments with n=4–8 mice per group. Graphs show mean value + SD. *p≤0.05, ***p≤0.001.

Following the immune cells characterization, we addressed the alterations in gut permeability in lupus mice. *Bank1^-/-^* IMQ-treated mice exhibited a reduction in gut epithelial barrier permeability compared to IMQ-treated WT mice (**Figure 2A**). Similarly, gut permeability was comparable between WT and TLR7Tg.*Bank1* ^-/-^, while TLR7Tg mice had increased permeability compared with WT mice (**Figure 2B**). Lupus mice also showed an altered ileal epithelium localization of the tight junction protein claudin-1. In both IMQ-treated WT mice and TLR7Tg mice, the distribution patterns of claudin-1 occurred in the form of aggregates within the cytoplasm rather than at the cellular membrane (**Figures 2C, D**), implying a compromised intestinal barrier. On the contrary, in both IMQ-treated *Bank1* ^-/-^ and TLR7Tg.*Bank1* ^-/-^ mice, claudin-1 was distributed similarly at the cell membrane as in WT naive mice (**Figures 2C, D**). This finding was consistent with the observed reduction in *in vivo* gut paracellular permeability in these mice.

The increased intestinal permeability, concomitant with inflammation, resulted in a differential translocation of commensal bacteria into nearby lymph nodes. Diverse genera, such as *RFN20*, along with *Rickettsia, Stenotrophomonas genic*, and *Lactobacillus salivarius* were identified in the mLN of TLR7Tg mice. Conversely, exclusively taxa from Enterobacteriaceae family were found to translocate into the mLN of TLR7Tg.*Bank1* ^-/-^ mice (**Figure 2E**), suggesting that the differential bacterial translocation may be attributable to alterations in the gut microbiota composition in the context of an increased gut permeability state.

### *Bank1* regulates gut IgA production and the IgA bacterial binding

**Figure 3:**
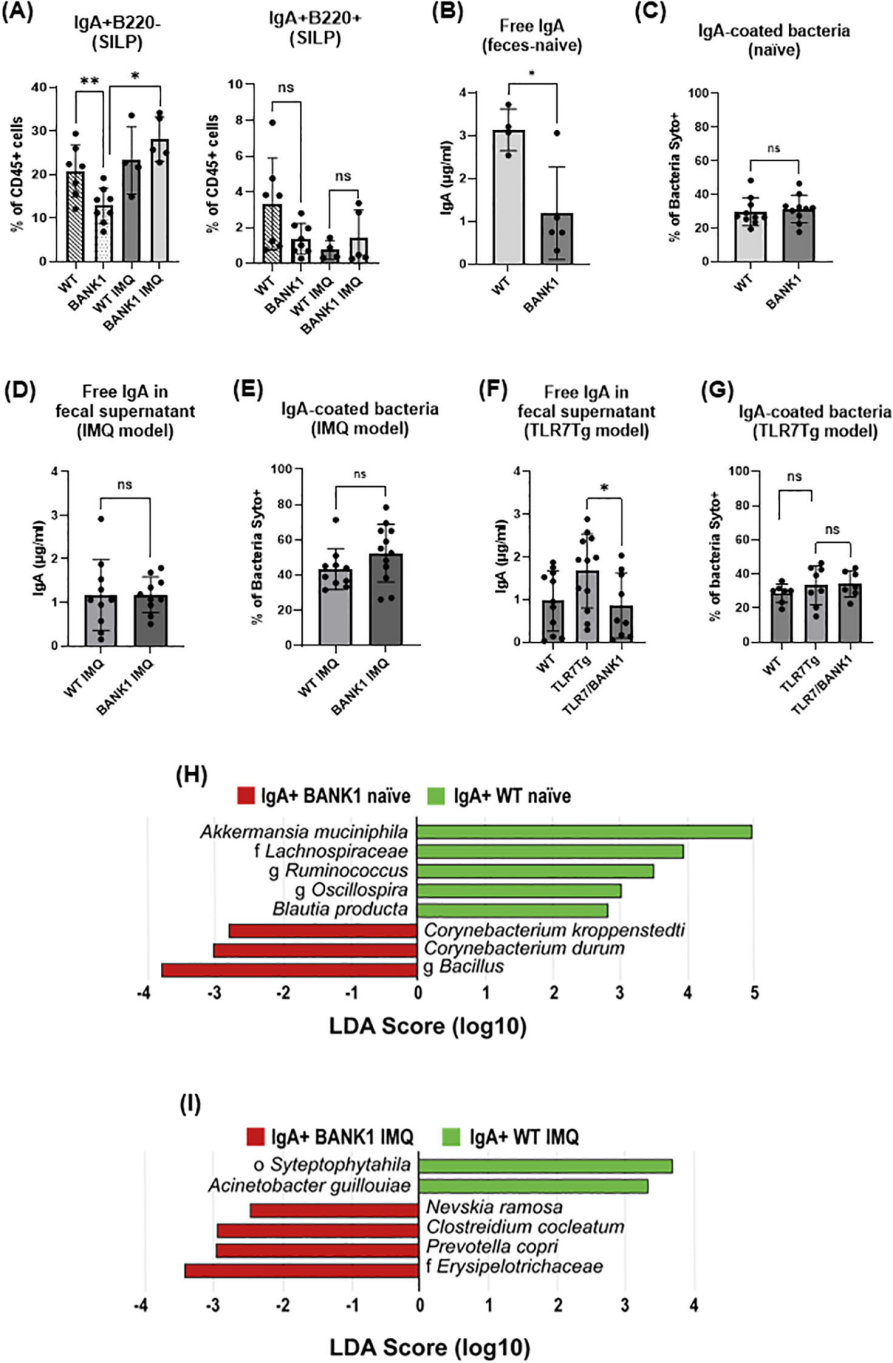
Proportions and levels of IgA^+^ B cells, secreted IgA, and IgA-bound bacteria in the presence or absence of Bank1 in lupus. **(A)** Frequencies of IgA^+^B220^+^ and IgA^+^B220^-^ cells (gated from the CD45^+^CD11c^-^F4/80^-^ cell population) in the SILP from naïve and IMQ-treated WT and *Bank1* ^-/-^mice. **(B)** Concentration of free IgA in fecal supernatants and **(C)** frequency of IgA-coated fecal bacteria from naïve WT and *Bank1* ^-/-^mice. **(D)** Levels of free IgA in fecal samples and **(E)** frequency of IgA-coated fecal bacteria from IMQ-treated WT and *Bank1* ^-/-^ mice. **(F)** Free fecal IgA and **(G)** IgA-coated fecal bacteria in mice from TLR7Tg model. **(H)** LEfSe plot of taxa found in the IgA^+^ fraction of fecal samples from naïve mice. **(I)** LEfSe plot of taxa found in the IgA^+^ fraction of fecal samples from IMQ-treated mice. Statistical analysis performed by Mann-Whitney unpaired test. Data represents results from 3–5 independent experiments with n=6–11 mice per group. In each graph is depicted the mean value with SD. *p≤0.05, **p≤0.01.

We next evaluated the changes in the IgA immune response and found that there was a significant reduction in the frequency of IgA^+^B220^-^ plasma cells in the SILP of naïve *Bank1^-/-^* mice (**Figure 3A**; **Supplementary Figure S2A**) compared with naïve WT controls. Although IgA^+^B220^+^ B cells showed a trend towards reduction in naïve *Bank1^-/-^* SILP, it did not reach significance (**Figure 3A** right). In contrast, both B220^-^ IgA^+^ and B220^+^ IgA^+^ cells accumulated in the PPs of *Bank1^-/-^* mice in comparison to WT controls (**Supplementary Figure S2B**). To more accurately address the role of *Bank1* on IgA production, isolated B cells from spleen and PPs of both naïve *Bank1^-/-^* and WT mice were stimulated *in vitro* with TGF-β, retinoic acid, anti-CD40 and anti-IgM to induce IgA^+^ differentiation. After stimulation, over 80% of the WT B cells, but only 62% of the *Bank1^-/-^* B cells, differentiated into IgA^+^ B cells. In contrast, no difference was observed with spleen B cells (**Supplementary Figure S2C**).

In accordance with the cellular results, lower levels of fecal free-IgA were found in naive *Bank1^-/-^* than in naive WT mice (**Figure 3B**); however, the frequency of fecal IgA-coated bacteria was not different between *Bank1^-/-^* and WT mice (**Figure 3C**). In lupus mice, the analysis of intestinal IgA production showed that IMQ-treated *Bank1* ^-/-^ mice had similar levels of fecal free IgA as IMQ-treated WT controls (**Figure 3D**). The IgA response to fecal bacteria, assessed as the frequency of IgA-coated bacteria, exhibited a slight, though not statistically significant, increase in IMQ-treated *Bank1^-/-^* mice compared with IMQ-treated WT controls (**Figure 3E**). Conversely, the levels of fecal free IgA were reduced in TLR7Tg.*Bank1* ^-/-^ compared with TLR7Tg mice (**Figure 3F**), nevertheless the frequency of IgA-coated bacteria was comparable between TLR7Tg and TLR7Tg.*Bank1* ^-/-^ mice (**Figure 3G**).

Given that the intestinal IgA response was altered in *Bank1^-/-^* mice, yet the frequency of IgA-coated bacteria remained comparable between *Bank1^-/-^* and WT mice both in steady state and during lupus, we reasoned that the binding pattern of the gut-secreted IgA to fecal bacteria might diverge between *Bank1^-/-^* and WT mice. To test this hypothesis, fecal bacteria were sorted according to their IgA coverage followed by their 16S rRNA gene sequencing. In naïve mice, within the IgA-coated bacteria, *Akkermansia muciniphila*, and the Lachnospiraceae family were most abundant in naïve WT mice, while in naive *Bank1^-/-^* mice, the genus *Bacillus* and *Corynebacterium* were the dominant taxa (**Figure 3H**). In IMQ-treated *Bank1* ^-/-^ mice, the most abundant IgA-coated bacterial taxa were the family Eryspelotrichaceae, *Prevotella copri*, *Clostridium cocleatum*, and *Nevskia ramosa*, while the order Streptophytahila and the species *Acinetobacter guillouiae* were the most abundant in the IgA-positive fraction of IMQ-treated WT (**Figure 3I**). In contrast, in the non-IgA-coated bacteria, IMQ-treated WT mice exhibited differential abundance of taxa from the family Gaiellacea, genera *Actinomyces*, *Streptococcus*, and *Corynebacterium*, as well as *Haemophilus parainfluenzae* (**Supplementary Figure S2D**). These results indicate that *Bank1* absence modifies the gut immune response and microbiota composition.

### *Bank1* regulates the microbiome composition of lupus mice

**Figure 4:**
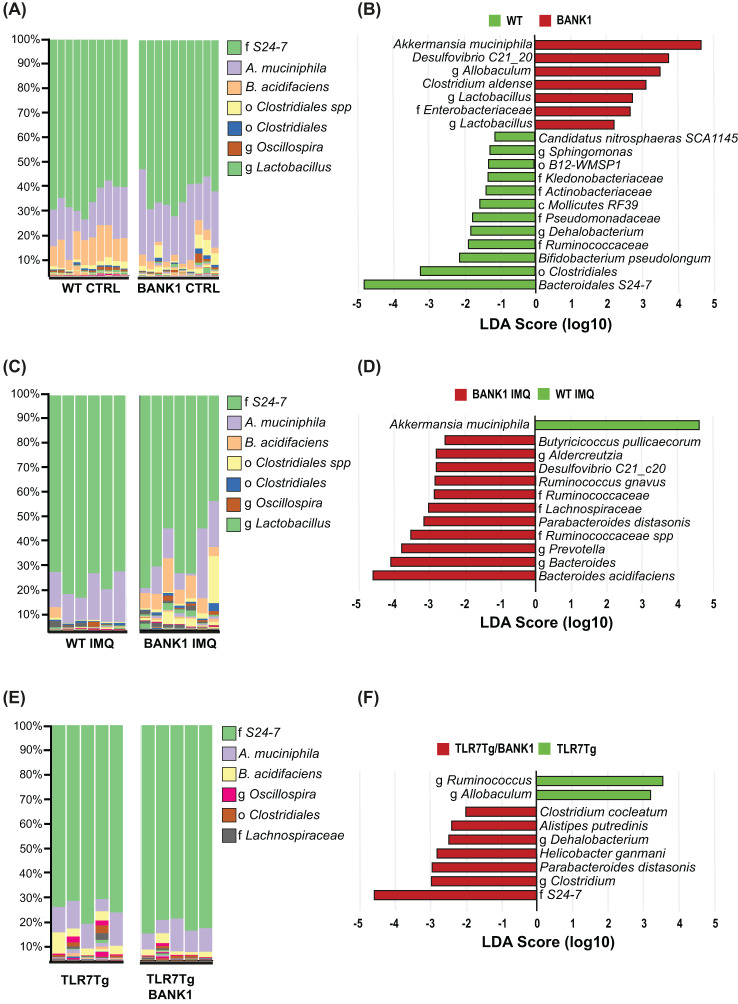
Bank1 modifies the gut microbiota composition regardless of disease status. **(A)** Bar-plots of taxa´s relative frequencies in the gut microbiome in naïve WT and *Bank1^-/-^* mice, **(C)** IMQ-treated WT and IMQ-treated *Bank1^-/-^* mice, and **(E)** TLR7Tg and TLR7Tg.*Bank1^-/-^* mice. **(B)** LEfSe plot of taxa found in the gut microbiome from naïve WT and *Bank1^-/-^* mice, **(D)** IMQ-treated WT and *Bank1^-/-^* mice, and **(F)** from the TLR7Tg lupus model. Each column in **(A)**, **(C)** and **(E)** represents one individual sample. Results are representative of 3 independent experiments.

When analyzing the gut microbiome composition of the various mouse strains, a significant difference was observed between naïve *Bank1^-/-^* and WT samples in alpha diversity (**Supplementary Figure S3A**), indicating changes in their richness, while in general, abundance of individual taxa was similar. A principal component analysis (PCA) and Bray-Curtis dissimilarity analysis (**Supplementary Figure S3B**) demonstrated that the beta diversity from gut microbiomes of naïve *Bank1^-/-^* and naïve WT mice exhibited a significant degree of dissimilarity (p=0.001). **Figure 4A** depicts the relative abundance of most abundant taxa in the *Bank1^-/-^* and WT fecal microbiomes. A LEfSe analysis revealed that *Akkermansia muciniphila* and *Desulfovibrio C21_C20* were the dominant taxa in *Bank1^-/-^* mice, whereas in WT mice, family *Muribaculaceae* (formerly known as S24-7), the order *Clostridiales*, *Bifidobacterium pseudolongum*, and family *Ruminococcaceae* were significantly more abundant (**Figure 4B**).

Also, the relative abundance of taxa revealed a switched bacterial ecology in IMQ-treated *Bank1* ^-/-^ from that of IMQ-treated WT mice (**Figure 4C**). The changes in taxonomic relative abundance were also observed in TLR7Tg mice when compared with TLR7Tg *Bank1* deficient mice (**Figure 4E**), but the changes observed in each lupus model were different. In IMQ-treated WT mice, *Akkermansia muciniphila* was the most abundant taxon, whereas *Bacteroides acidifaciens*, *Parabacteroides distasonis*, *Ruminoccocus gnavus*, *Desulfovibrio C21_20*, and *Butyricicoccus pullicaecorum* were the most abundant species in the IMQ-treated *Bank1* ^-/-^ mice (**Figure 4D**). In the TLR7Tg lupus mice, the most abundant genera were *Ruminococcus* and *Allobaculum*, while in the TLR7Tg.*Bank1* ^-/-^ the most abundant family was *Muribaculaceae*, together with *Parabacteroides distasonis*, *Helicobacter ganmani*, *Alistipes putrideni*s, and *Clostridium cocleatum* species (**Figure 4F**). The beta diversity analysis of IMQ-treated *Bank1^-/-^* and IMQ-treated WT mice also showed to be significantly different (p=0.011) (**Supplementary Figure S3C**). Furthermore, the ANCOM analysis showed that *P. distasonis* and *B. acidifaciens* were common bacteria in the gut of *Bank1* ^-/-^ mice with lupus both in the IMQ-treated (**Supplementary Figure S3D**) and in the TLR7Tg.*Bank1* ^-/-^ mice (**Supplementary Figure S3E**). These findings were validated by qPCR of cultured *P. distasonis* and *B. acidifaciens* bacteria (**Supplementary Figure S3F**). This evidence supports the key role of *Bank1* in shaping the gut bacterial composition.

### Cecal metabolite relationship with gut bacteria in lupus mice deficient for *Bank1*

**Figure 5:**
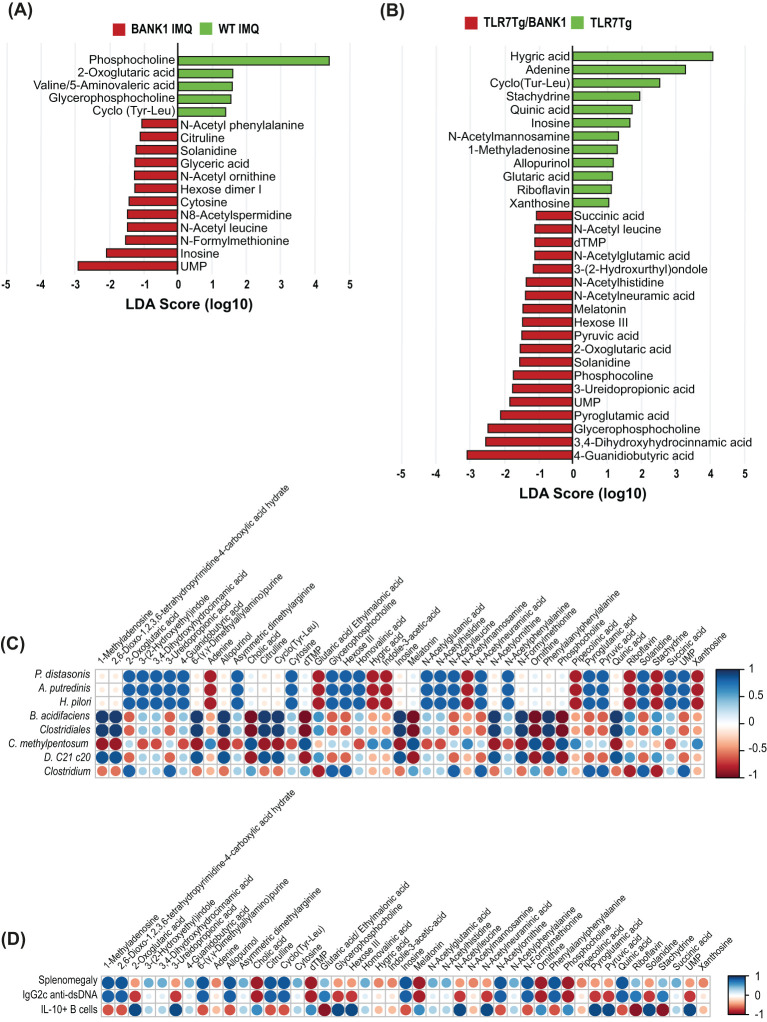
Bank1 modifies the cecal metabolite production in lupus. **(A)** LEfSe plot of cecal metabolites in samples from IMQ-treated WT and *Bank1^-/-^* mice and **(B)** from TLR7Tg and TLR7Tg.*Bank1^-/-^* mice. **(C)** Correlation analysis of the significantly different bacterial species in fecal samples and metabolites’ abundance in cecal samples in lupus WT and *Bank1^-/-^* mice. **(D)** Correlation analysis of the cecal metabolites and splenomegaly, IgG2c anti-dsDNA, and IL-10 producing B cells. Cecal and fecal samples were collected from 5–8 mice of each genotype.

To further characterize the microbiome-related functions in the gut immune response, a metabolomic characterization was conducted in the cecal content as a reflectION of bacterial metabolism in the gastrointestinal tract. The cecal content of IMQ-treated *Bank1* ^-/-^ mice showed a higher abundance of uracyl monophosphate (UMP), inosine, N-formylmethionine, and N-acetyl leucine compared with IMQ-treated WT mice. In contrast, phosphocoline and 2-oxoglutaric acid were enriched in the cecal content of IMQ-treated WT mice (**Figure 5A**). Whilst 4-guanidino butyric acid, UMP, and N-acetyl leucine were more abundant in TLR7.*Bank1* ^-/-^ mice, hygric acid and adenine were more abundant in TLR7Tg mice (**Figure 5B**). UMP, N-acetyl-leucine, and solanidine were found to be highly abundant in both IMQ-treated *Bank1^-/-^* mice and TLR7.*Bank1^-/-^*.

A correlation analysis of the most abundant fecal bacteria with cecal metabolites demonstrated positive correlations between *P. distasonis* and the abundance of UMP, N-acetyl leucine, and solanidine (**Figure 5C**), which are closely linked to the absence of *Bank1*. On the contrary, these same metabolites showed a negative correlation with splenomegaly and serum levels of IgG2c anti-dsDNA antibody levels, and a positive correlation with the abundance of IL-10-producing B cells from the Peyer’s patches (**Figure 5D**). These results delimit a list of potential metabolites exhibiting immunomodulatory capacity.

### *Bank1* microbiome composition regulates lupus severity

**Figure 6:**
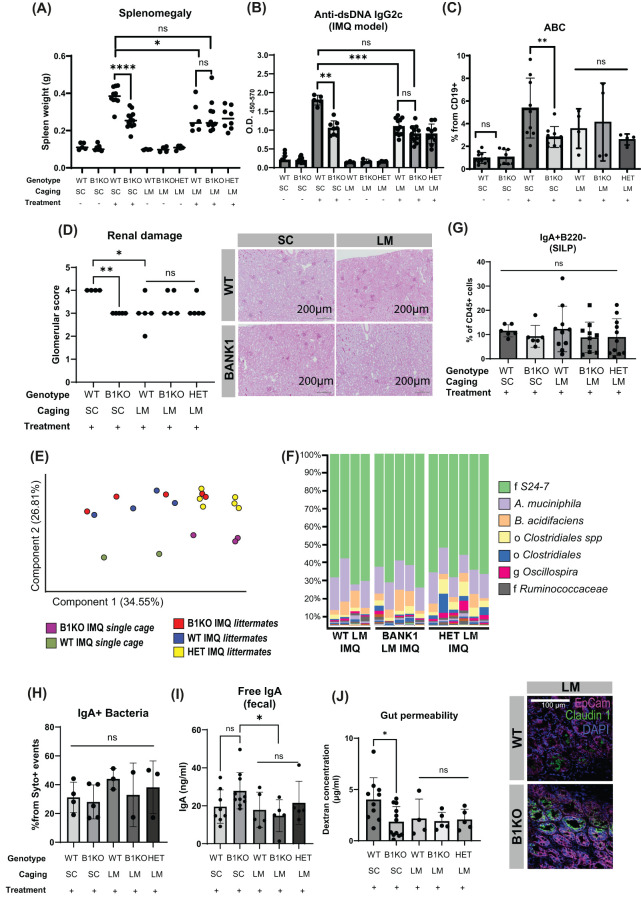
Bank1-associated microbiota limits lupus inflammation. **(A)** Spleen weight of single cage and littermate mice either naïve or with IMQ-induced lupus. **(B)** Anti-dsDNA IgG2c antibody titers in serum of littermate and single cage mice treated or not with IMQ. **(C)** ABC (CD11b^+^ CD11c^+^ T-bet^+^ gated from CD19^+^ B cells) frequency in the spleen of littermate and single cage IMQ-treated mice and from single cage naïve WT and *Bank1^-/-^* mice. **(D)** Glomerular damage scores and representative images of PAS staining in longitudinal sections from the kidney of IMQ-treated single cage and littermate mice. **(E)** PCA showing the distribution of the microbiome in IMQ-treated single cage and littermate mice. **(F)** Bar-plots of taxa´s relative frequencies found in the gut microbiome in IMQ-treated littermate mice. **(G)** Frequencies of IgA^+^B220^-^ B cells in the SILP, **(H)** quantification of free fecal IgA in the gut lumen and **(I)** Frequencies of IgA-coated fecal bacteria of IMQ-treated single cage and littermate mice. **(J)** *In vivo* assessment of gut permeability by oral administration of FITC-Dextran to IMQ-treated single cage and littermate mice. **(K)** Claudin-1 distribution in transversal sections of the ileal epithelium of IMQ-treated littermate mice. Statistical analysis performed by one way ANOVA. Data represents results from 3 independent experiments with similar results and n=5–10 mice per group. Graphs represent mean value with SD. SC: single cage. LM: Littermate. B1KO: *Bank1* ^-/-^, HET: *Bank1* ^+/-^, WT: *Bank1* ^+/+^ mice. *p≤0.05, **p≤0.01, ***p≤0.001, ****p≤0.0001.

We next wanted to address whether the gut microbiota supported by *Bank1* deficiency might play an active role in the ameliorated phenotype found in IMQ-treated *Bank1^-/-^* mice. To that end, littermate mice (*Bank1* ^-/-^, *Bank1* ^+/+^, and *Bank1* ^+/-^) carrying the *Bank1* ^-/-^ microbiota were generated and lupus was induced using the IMQ model. Microbiota-control mice were IMQ-treated WT and IMQ-treated *Bank1* ^-/-^ mice, which were bred in separate cages (single cage-mice) to maintain each genotype-associated gut microbiota separately throughout the whole experiment. The gut microbiota establishment in naïve littermate mice did not carry any significant autoimmune signs (**Figures 6A, B**). As expected, upon IMQ treatment, single-cage *Bank1^-/-^* mice developed less severe lupus compared with single-cage WT mice. On the contrary, the disease that manifested after IMQ-treatment in littermate mice was similar regardless of their genotype and similar to that observed in *Bank1* ^-/-^ single-cage mice, with same spleen size and serum levels of IgG2c and total IgG anti-dsDNA antibodies (**Figures 6A, B**; **Supplementary Figure S4A**). Similarly, the frequency of ABCs from the CD19^+^ population in the spleen, which was reduced in single cage IMQ-treated *Bank1^-/-^* mice compared with single cage IMQ-treated WT mice, was equivalent in littermate mice irrespective of their genotype (**Figure 6C**). The renal evaluation showed equal kidney damage across IMQ-treated littermate mice, and it was different from that of IMQ-treated WT single-cage mice, but not from IMQ-treated *Bank1* ^-/-^ single cage mice (**Figure 6D**).

Sequencing and analysis of the fecal microbiome of IMQ-treated littermates revealed a similar microbiome composition between littermate feces and divergent from both IMQ-treated WT and *Bank1^-/-^* single-cage mice, which were also different between them (**Figure 6E**). Likewise, the relative abundance of the most abundant taxa was similar across IMQ-treated littermates (**Figure 6F**). *P. distasonis* abundance, which was significantly increased in IMQ-treated single-cage *Bank1^-/-^* mice compared to their WT single-cage counterparts, showed no changes between IMQ-treated littermates and IMQ-treated single-cage *Bank1^-/-^* mice (**Supplementary Figure S4B**).

Analysis of the gut immune response revealed a reduction in the number of SILP B cells in naive littermate *Bank1^-/-^* mice when compared with naive littermate WT mice, whereas the recruitment of IgA^+^B220^-^ plasma cells was not (**Supplementary Figure S4C**). The differentiation of B cells to IgA^+^ plasma cells in the SILP was comparable across genotypes of IMQ-treated littermate mice (**Figure 6G**), as was the frequency of IgA-coated fecal bacteria (**Figure 6H**). The production of IgA was equivalent in single cage IMQ-treated *Bank1^-/-^* and WT mice, whereas IMQ-treated littermate *Bank1^-/-^* mice had reduced levels of free fecal IgA compared with IMQ-treated single cage *Bank1^-/-^* mice (**Figure 6I**). Furthermore, the sequencing of sorted fecal bacteria according to their IgA-coating showed that the genus *Lactobacillus*, and species *Actinobacter guillouiae*, and *Serratia marcescens* were the IgA-coated bacteria strains associated with IMQ-treated littermate WT mice and *A. muciniphila*, *Clostridium cocleatum*, and genus *Streptococcus* associated with IMQ-treated single cage WT mice (**Supplementary Figure S4D**). While IMQ-treated single cage *Bank1^-/-^* mice had *A. munciniphila*, family Enterobacteriaceae, and genus *Prevotella* as associated IgA^-^coated bacteria (**Supplementary Figure S4E**). Pearson’s correlation analysis of gut bacteria showed that *P. distasonis* had a negative correlation with splenomegaly, and anti-dsDNA IgG2c levels in serum, but a positive correlation with the PPs derived IL-10 producing B cells (**Supplementary Figure S4F**).

Finally, similar to single-cage IMQ-treated *Bank1^-/-^* mice, *in vivo* gut paracellular permeability was reduced in both WT and *Bank1^-/-^* IMQ-treated littermates mice compared with IMQ-treated single-cage WT mice (**Figure 6J**). The reduced gut permeability correlated with a cell membrane distribution of the gut epithelial tight-junction protein claudin-1 in the small intestine, which was comparable across IMQ-treated littermate mice and more similar to IMQ-treated single-cage *Bank1^-/-^* than to single-cage IMQ-treated WT mice (**Figure 6K**). These findings highlight the influence of gut microbiota composition in TLR7-mediated lupus severity.

### *P. distasonis* is associated with reduced gut barrier permeability and lupus inflammation

Given that *B. acidifaciens* and *P. distasonis* were among the most abundant species in the gut of *Bank1^-/-^* mice with lupus, we elected to further validate the microbial signature associated with the absence of *Bank1* in lupus by means of a Wilcoxon test and recursive feature elimination (RFE). Among the 104 taxa identified in the 16S sequencing, 66 taxa had a p-value less than 0.05. However, only *P. distasonis* was selected after RFE, indicating that only this taxon was significantly associated with the *Bank1^-/-^* genotype.

**Figure 7:**
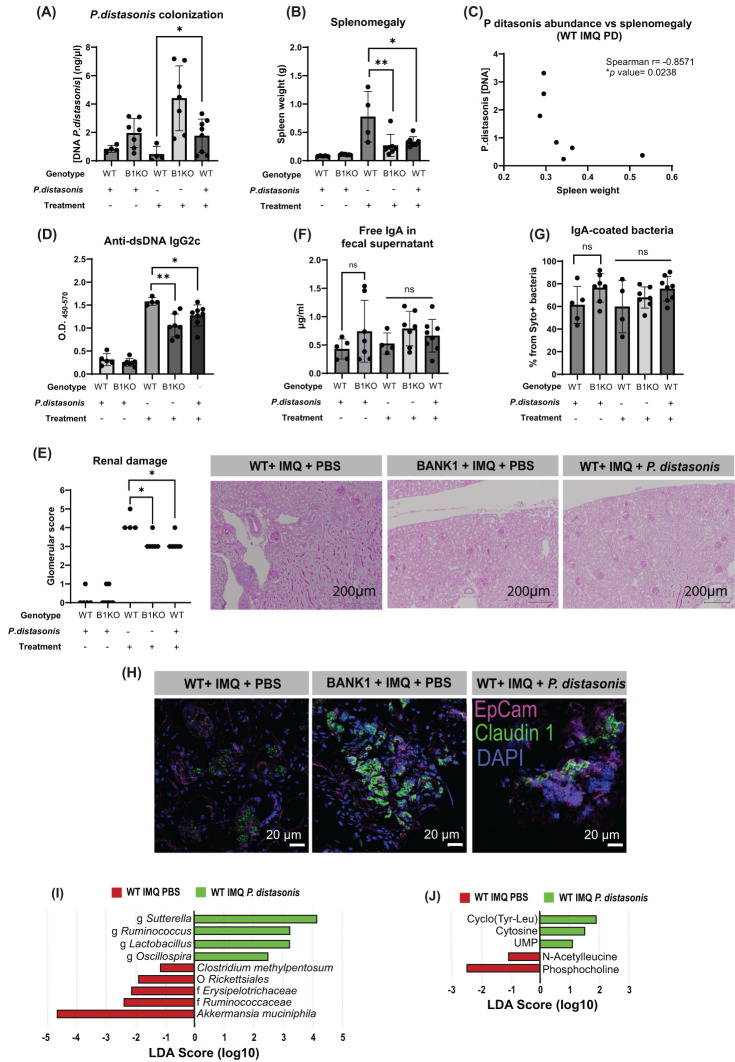
*Parabacteroides distasonis* modifies the gut microbiota and downregulates inflammation. **(A)** *P. distasonis* presence in fecal samples at the end point by qPCR **(B)** Spleen weight of mice that were administered *P.distasonis* either treated or not with IMQ. **(C)** Correlation between spleen weight and the abundance of P. distasonis in fecal samples. **(D)** IgG2c anti-dsDNA antibody titers in serum. **(E)** PAS-stained longitudinal kidney sections and renal damage characterized by glomerular score. **(F)** Fecal free IgA levels measured and **(G)** frequencies of IgA-covered bacteria. **(H)** Claudin-1 staining in longitudinal sections of the ileal epithelium; claudin 1- aF488 (green), EpCam-APC (pink), nuclei-DAPI (blue). **(I)** LEfSe plot of taxa found in fecal samples form IMQ-treated PBS-supplemented WT and IMQ-treated *P. distasonis*-supplemented WT mice. **(J)** LEfSe plot of cecal metabolites found in samples from IMQ-treated PBS-supplemented WT and IMQ-treated *P. distasonis*-supplemented WT mice. Statistical analysis performed by one way ANOVA with n=4–11 mice per group. Graphs represent mean value with SD. *p≤0.05, **p≤0.01.

Consequently, we proceeded to investigate whether modulating the gut microbiome by introducing *P. distasonis* could potentially attenuate the inflammatory response in lupus. The daily gastrointestinal supplementation of WT mice with *P. distasonis* resulted in increased amounts of these bacteria in the feces (**Figure 7A**), indicating that *P. distasonis* effectively populated mice intestines. *P. distasonis* supplementation did not induce changes in the spleen sizes of naive WT and *Bank1* ^-/-^ mice. IMQ-treated WT mice that received *P. distasonis* presented a significantly reduced splenomegaly compared with IMQ-treated WT mice receiving PBS, but comparable to that observed in IMQ-treated *Bank1* ^-/-^ mice receiving PBS (**Figure 7B**). Also, we observed a negative correlation between *P. distasonis* fecal levels and the spleen weight in IMQ-treated WT mice receiving *P. distasonis* (**Figure 7C**), but there was no such correlation in IMQ-treated PBS-gavaged WT mice (**Supplementary Figure S5A**).

IMQ-treated WT mice receiving *P. distasonis* had reduced levels of IgG2c anti-dsDNA (**Figure 7D**). Accordingly, the less severe form of lupus in IMQ-treated WT mice gavaged with *P. distasonis* was concomitant with lessened renal damage (**Figure 7E**).

The activation of B cells in the PPs was downregulated in naive mice that received *P. distasonis*, having *Bank1* ^-/-^ mice a lower frequency of GC B cells compared with naive WT mice (**Supplementary Figure S5B**). Upon IMQ treatment, there were no differences in the frequencies of GC B cells between the WT mice gavaged with *P. distasonis* and *Bank1* ^-/-^ gavaged with PBS, but these frequencies were significantly reduced compared with IMQ-treated WT mice that received PBS (**Supplementary Figure S5B**). In the spleen, as expected, there was a reduction in the number of GC B cells in the IMQ-treated *Bank1* ^-/-^ PBS-gavaged compared with IMQ-treated PBS-gavaged WT mice. Unexpectedly, an increased frequency of GC B cells was observed in IMQ-treated WT mice that received *P. distasonis* compared with IMQ-treated *Bank1* ^-/-^ PBS gavaged mice (**Supplementary Figure S5C**).

The frequency of IgA-producing cells in the SILP was not different in naive and IMQ-treated mice, regardless of whether they received *P. distasonis* or PBS (**Supplementary Figure S5D**). Whereas, in the PPs of naïve *Bank1^-/-^* mice gavaged with *P. distasonis*, the frequency of IgA-producing cells was significantly reduced (**Supplementary Figure S5E**). Supplementation with *P. distasonis* did not alter either the presence of free IgA in feces (**Figure 7F**) nor the level of IgA-coverage of fecal bacteria (**Figure 7G**).

WT mice that received *P. distasonis* showed cell membrane localization of claudin-1 within the gut epithelium, contrary to the cytoplasm vesicle-like localization of claudin-1 in WT mice PBS-gavaged (**Figure 7H**).

The analysis of the microbiome of mice gavaged with *P. distasonis* showed that the richness and evenness of the microbiota of IMQ-treated *P. distasonis*-supplemented WT mice and IMQ-treated PBS-control WT mice were comparable (**Supplementary Figure S5F**). On the contrary, the beta diversity analysis showed significant differences in gut microbiota composition between WT *P. distasonis*-supplemented WT mice and PBS-gavaged WT mice with lupus. (**Supplementary Figure S5G**). There were also changes in the relative abundance of bacteria taxon between IMQ-treated WT mice that received PBS and IMQ-treated WT mice that received *P. distasonis* (**Supplementary Figure S5H**). In WT mice treated with IMQ and gavaged with PBS the most abundant taxon were *Akkermansia muchiniphila* and *Clostridium methylpentosum* species, whereas in WT mice treated with IMQ and gavaged with *P. distasonis* the most abundant taxon were *Sutterella*, *Ruminococcus*, Lactobacillus, and *Oscillospira* genera (**Figure 7I**).

The metabolite production analysis of the cecal bacteria showed that UMP, cytosine, and cyclo(Tyr-Leu) metabolites where highly abundant in IMQ-treated WT mice that received *P. distasonis*, whereas phosphocholine and acetyl leucine were more abundant in IMQ-treated WT PBS-gavaged mice (**Figure 7J**). These results highly resemble those obtained when comparing IMQ-treated single cage WT and *Bank1^-/-^* mice, which strongly suggest an immunomodulatory effect of *P.distasonis* in lupus pathogenesis.

In order to further characterize the effects of *P. distasonis*, total B cells from PPs of naïve mice were cultured for 48 hours in the presence of heat-inactivated bacteria. The frequency of differentiated IgA^+^ B220^+^ cells found in Bank1^-/-^ cultures was lower compared with WT cultures (see **Supplementary Figure S5I** left). In accordance with prior results, co-culture of *P. distasonis* with splenic total B cells revealed no differences in differentiated IgA^+^ B220^+^ cell frequency between Bank1^-/-^ and WT animals (**Supplementary Figure S5I** right).

### *P. distasonis* and CpG stimuli induce IL-10 in Peyer’s patches B cells

**Figure 8:**
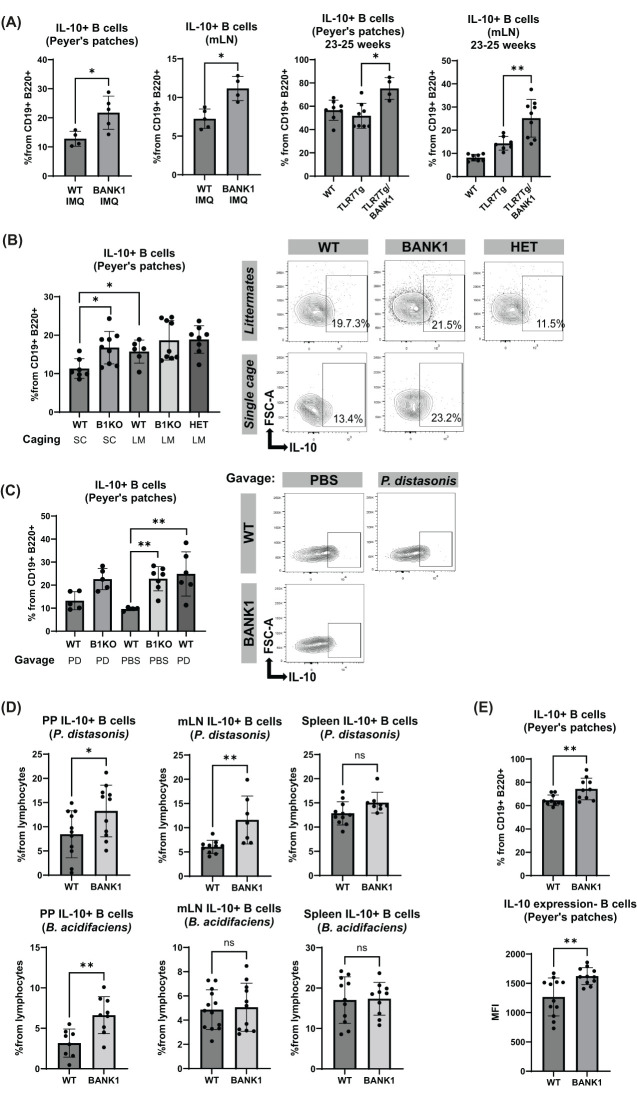
IL-10 production by PPs and mLN B cells increases with the presence of *P. distasonis*. **(A)** Frequency of IL-10^+^ B cells characterized by flow cytometry in PPs and mLN from IMQ-treated mice and mice from the TLR7Tg model. **(B)** IL-10^+^ B cells levels in the PPs from IMQ-treated single cage and littermate mice and representative plots. **(C)** IL-10^+^ B cells in PPs from IMQ-treated and naïve, PBS- or *P. distasonis*-gavaged mice. **(D)** B cells were purified from PPs, mLN, and spleen to be stimulated either with P. distasonis or *B*. *acidifaciens* for 48 hours to determine the IL-10^+^ B cells levels by flow cytometry. **(E)** IL-10 producing B cells frequencies and IL-10 MFI after CpG stimulation of B cells isolated from the PPs. Statistical analysis performed by Mann-Whitney unpaired test. Data representative from 2–3 independent experiments with 5–11 mice per group. Graphs represent mean value with SD. *p≤0.05, **p≤0.01.

To characterize the mechanism by which gut microbiota could be regulating lupus manifestations, we then analyzed the frequency of IL-10-producing B cells. The *ex vivo* measurement of this population showed that B cells from the PPs and mLN of both IMQ-treated *Bank1^-/-^* andTLR7Tg.*Bank1* ^-/-^ mice had a higher frequency of IL-10^+^ B cells than B cells from their IMQ-treated WT or TLR7Tg mice counterparts, respectively (**Figure 8A**; **Supplementary Figure S6A**). This was not observed in the spleen-derived B cells, where the IL-10 production was similar between B cells from *Bank1^-/-^* and WT lupus mice (**Supplementary Figure S6B**). Expression levels (MFI) of IL-10 in B cells from PP were comparable between IMQ-treated *Bank1^-/-^* and IMQ-treated WT mice (**Supplementary Figure S6C**), whereas in TLR7Tg.*Bank1^-/-^* B cells, the IL-10 had higher MFI in PP and mLN compared with TLR7.Tg B cells (**Supplementary Figure S6D**).

To evaluate the effect of microbiota composition in IL-10 production, we determined its production in PPs B cells of IMQ-treated littermates and found that, along with gut microbiota vertical transfer, the production of IL-10 was also equal, regardless of the genotype of all littermate mice (**Figure 8B**). IL-10 MFI was comparable as well (**Supplementary Figure S6E**). The levels of IL-10 production by B cells of IMQ-treated littermates were comparable to that observed in IMQ-treated single cage *Bank1* ^-/-^ mice (**Figure 8B**).

We also examined the IL10^+^ B cells in the PPs of WT mice that were gavaged with *P. distasonis* and treated with IMQ. We found that *P. distasonis* supplementation in IMQ-treated WT mice induced significantly higher percentages of IL-10 secreting B cell from PPs than IMQ-treated WT mice that received PBS (**Figure 8C**). On the contrary, the frequency of IL-10 secreting B cells from PPs of IMQ-treated WT mice gavaged with *P. distasonis* was similar to that of PBS-gavaged IMQ-treated *Bank1^-/-^* mice. A slight increase, but not significant, in IL-10 MFI was observed in IMQ-treated *P. distasonis*-gavaged WT mice compared with IMQ-treated PBS-gavaged WT mice (**Supplementary Figure S6F**). Similarly, the mLN showed a nonsignificant trend towards increased production of IL-10 by B cells in IMQ-treated *P. distasonis*-gavaged WT mice in comparison to IMQ-treated PBS-gavaged WT mice (**Supplementary Figure S6G**).

Because *P. distasonis* and *B. acidifaciens* were differentially more abundant in *Bank1* ^-/-^ mice upon lupus development, we assessed their immunoregulatory capacity. *Bank1* ^-/-^ B cells from PPs cultured with either *P. distasonis* or *B. acidifaciens* produced significantly higher amounts of IL-10 than WT B cells (**Figure 8D**), while in mLN, an increase in IL-10 production was only observed in *Bank1* ^-/-^ B cells stimulated with *P. distasonis*, but not *B. acidifaciens* (**Figure 8D**). No differences were observed in IL-10 production in spleen B cells when stimulated with each bacteria strain (**Figure 8D**, right).

Finally, purified B cells from PPs, mLN, and spleen were stimulated *in vitro* with CpG. This stimulation resulted in higher production of IL-10 by *Bank1* ^-/-^ B cells compared with WT B cells from PP, but not in B cells from the spleen or the mLN (**Figure 8E**; **Supplementary Figure S6H**). Additionally, *Bank1* ^-/-^ deficient B cells from PPs presented a higher IL-10 MFI compared with their WT counterparts. This was not the case for IL-10 in mLN nor spleen B cells (**Figure 8E**; **Supplementary Figure S6I**).

### The microbiome of lupus patients is devoid of *P. distasonis*

**Figure 9:**
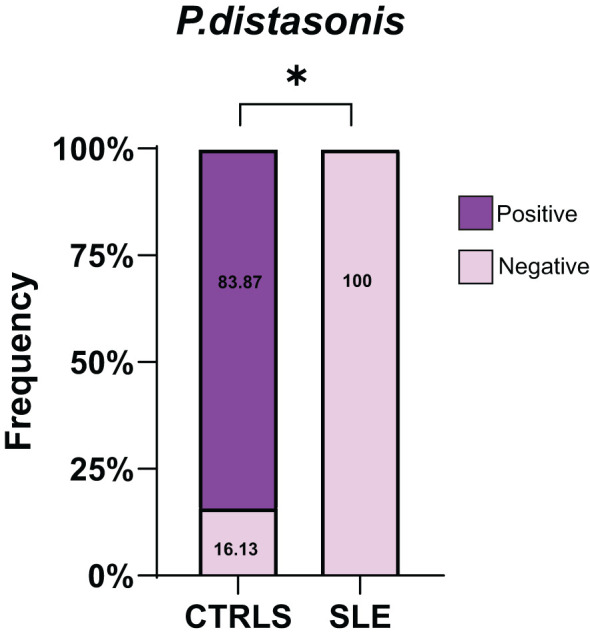
Absence of *P. distasonis* from the microbiome of lupus patients. Frequency of individuals positive for *P. distasonis* in blood microbiome after sequencing the V4 region of 16S rRNA gene.

Lastly, we analyzed by 16s rRNA sequencing the blood microbiome of an available cohort of lupus patients and compare it with the profile found in healthy controls. Consistent with our observations in the lupus mouse models, *P. distasonis* was undetectable in all blood samples of lupus patients (n=26), while 16.1% of healthy control samples were positive for *P. distasonis* (n= 31) (**Figure 9**). Characteristics of lupus patients and healthy controls are shown in **Table 2**.

**Table 2:** Gender, age, and treatment of lupus patients and healthy controls.

Subjects	Sex	Age (years)	Immunosuppressants	Steroids
Lupus	Women 96% (n=25)	48.92	19%	46%
	Men 4% (n=1)	47		
Healthy Controls	Women 74% (n=23)	46.9	N/A	N/A
	Men 26% (n=8)	42.8		

## Discussion

We aimed at investigating how *Bank1* regulates the intestinal immune response in the context of lupus development previously observed to be immunomodulated by its absence (20, 21). Our analysis of the intestinal B cell response revealed that *Bank1* deficiency was associated with an altered IgA response and microbiota changes in both steady state and disease. During the analysis of gut inflammation parameters in lupus*, Bank1* ^-/-^ mice microbiota showed a persistent increase of *P. distasonis* within the microbial community that correlated with dampened lupus severity. Similar results were observed with the vertical transfer of *Bank1-*like microbiota to WT littermate mice and the oral supplementation of IMQ-induced WT mice with *P. distasonis*, reducing significantly lupus endpoint manifestations. These effects were accompanied with decreased intestinal permeability, increased IL-10-producing B cells in the PPs and increased cecal metabolites known to induce the expression of tight junction proteins in the gut epithelium (35).

Absence of *Bank1* impaired the recruitment of B cells to the SILP and was indispensable for GC B cell proliferation in PPs, yet dispensable for B cells and IgA^+^ B220^-^ cells differentiation and aggregation in the SILP and PPs. However, continuous activation of TLR7 in the gut enhanced the production of IgA, both in PPs and the SILP, thereby increasing the levels of secreted IgA into the lumen with potential commensal bacteria coating and remodeling of the microbial community colonization (36). Because TLR7 activation induces a lupus-like autoimmune disease, the mucosal injury and alterations reported in IMQ-treated and TLR7Tg mice are direct manifestations of the gut-associated damage resulting from the autoimmunity process.

IgA-class switching induced by the *in vitro* activation including *P. distasonis* was impaired in *Bank1^-/-^* PPs but not in *Bank1^-/-^* spleen B cells. This disparate response of B cells from different anatomical sites may be related to molecular and structural dissimilarities in the surrounding milieu. Mucosal B cells are exposed to constant activation signals from commensals microbials and are prone to respond differently than B cells located far from the intestinal firewall (37, 38). Although there were defects in IgA secretion in the absence of *Bank1*, IgA coated bacteria showed a similar frequency as controls, albeit with differential bindings. This observation was consistent with what has been reported for Bruton’s tyrosine kinase (*Btk*) deficient mice (39), another B cell signaling molecule involved in TLR activation, where the intestinal microbiota ecology was modified as well as the gut-secreted IgA specificity. Interestingly, the IgA-based sequencing of fecal bacteria demonstrated that the IgA binding to gut bacteria changed in the absence of *Bank1* in mice raised in separate cages. However, the homogenization of the microbiota also induced changes in the bacterial IgA-binding in WT mice, indicating that the microbiota composition also drives the IgA binding.

Like our results of the microbiome analysis, it has been reported that the triple lupus congenic B6.*Sle1.Sle2.Sle3* and B6.TLR7Tg lupus prone-mice, showed no changes in alpha diversity when compared with their control counterparts, but marked alterations in specific taxa composition (27, 40). Particularly, we found that the absence of *Bank1* allowed the increase of *P. distasonis* abundance during lupus development in both the IMQ-induced model and the TLR7Tg model.

Gut microbiota profiling in patients with rheumatoid arthritis, multiple sclerosis, Alzheimer disease, obesity, and nonalcoholic fatty liver has revealed a decreased abundance of *P. distasonis*. However, its association with lupus has not been determined. Furthermore, a fecal microbiome signature in lupus has not been identified (41). Therefore, we decided to analyze the plasma microbiome as it offers a more direct connection to systemic processes. Mirroring our mouse findings, lupus patients had undetectable levels of *P. distasonis* in their blood.

*P. distasonis* has been shown to possess anti-inflammatory properties (42–44). In our experiments, supplementing *P. distasonis* in the IMQ-induced WT mice, phenocopied the diminished lupus severity observed in *Bank1*-deficient mice. Furthermore, the reduced severity of the disease in littermate mice, regardless of their genotype, but with homogenized microbiota that includes *P. distasonis* presence, indicates that the microbiome is a crucial environmental factor that has the capacity to modulate disease development. However, the specific microbiome composition that emerges upon autoimmune inflammation depends on the genetic background of the mice. For instance, *P. distasonis* was shown to had a detrimental effect in autoimmune diabetes due to the presence of antigens that mimic beta-cell antigens (45), whereas the combination of *P. distasonis* and *A. muciniphila* reduced the severity of colitis (46).

In our lupus model mediated by TLR7-signaling, the deficiency in *Bank1* gene particularly allowed an increase in the abundance of *P. distasonis* during lupus inflammation development and *P. distasonis* was proved to exert anti-inflammatory effects. In murine lupus models, *L. reuteri*, *Lactobacillus* spp, *A. muciniphila* and *L. plantarum* have been reported to modulate gut permeability and dysbiosis consequently ameliorating disease severity (27, 47, 48). The enrichment of *P. distasonis* in lupus mice conserved claudin-1 localized to the epithelial cell membrane and was associated with reduced gut permeability and dampened systemic inflammation. Additionally, the *Bank1* deficiency and the proliferation of *P. distasonis*, allowed the differential translocation of commensals, restraining that of *Lactobacillus salivarius*, which has been positively correlated with the disease activity in lupus patients (49).

Lupus patients present a significantly different bacterial-derived metabolic pattern in their gut compared with healthy controls (50). The cecal content of IMQ-induced WT mice receiving *P. distasonis* was enriched in UMP, and the cecal content of *Bank1^-/-^* mice with lupus was enriched in inosine and UMP. These metabolites induce the expression of tight junctions in gut epithelial cells (51). Additionally, UMP has been shown to increase the apoptosis ratio of intestinal epithelial cells, thereby facilitating the renewal of intestinal villi tips (35). These two phenomena might contribute to the reduction of intestinal permeability observed in *Bank1^-/-^* mice with lupus.

In lupus, the deficiency in IL-10-producing B cells has been related to worse disease outcomes (14, 15). In *Bank1^-/-^* mice, the increased production of IL-10 by PPs B cells seems to be related to the presence of *P. distasonis*, as PPs-derived B cells from IMQ-treated WT littermates and WT mice supplemented with *P. distasonis* showed increased IL-10 expression *ex vivo*. Additionally, upon either bacterial or CpG stimulation of *Bank1*-deficient B cells from PPs, but not from spleen, there was increased production of IL-10, suggesting that the gut *Bank1*-microbiota might prime PPs-associated B cells into a regulatory-like phenotype. Importantly, membrane receptors expressed by B cells in the constant presence of specific microorganisms and/or their molecular and metabolic markers in the gut differs from that of B cells from other locations (52). In line with our findings, it has been described that in lupus patients, CD19^+^CD24^hi^CD38^hi^ B cells exhibited reduced regulatory capacity and reduced IL-10 secretion (14). Furthermore, the expression of *Bank1* has been reported to be reduced in these regulatory B cells. Likewise, human regulatory B cell subpopulations, such as IL-10-producing B cells and Granzyme^+^ B cells express low levels of *BANK1* (12), supporting a regulatory role of *Bank1* in B cell differentiation.

Genetic variants of lupus susceptibility account only for a portion of the observed variation in the phenotype that cannot explain the complexity of its pathophysiology and different clinical manifestations. The gut microbiome could be another key determining factor. The genetics of the host and the gut microbiota may be interdependent in triggering singular autoimmunity cascade effects, which could better explain the varying effects caused by a specific taxon in different host genetic backgrounds (53, 54).

## Data Availability

The original contributions presented in the study are included in the article/**Supplementary Material**, further inquiries can be directed to the corresponding author/s.

## Supplementary material

Supplementary Figure 1
*Bank1* dampens the gut and systemic inflammation induced by TLR7. **(A)** Assessment of CD19+B220+ B cells in the mLN of naïve and IMQ-treated WT and Bank1-/- mice. **(B)** Representation of the gating strategy for GC B cells frequency analysis. **(C)** GC B cells frequency in the spleen of IMQ-treated WT and *Bank1-/-* mice and mice from the TLR7Tg model. **(D)** Spleen weigh of the IMQ model (left) and TLR7Tg model (right). **(E)** anti-dsDNA IgG2c antibodies in serum in IMQ-treated mice (left) and TLR7Tg lupus model (right). **(F)** Representative images of the most common renal lesions found in TLR7Tg (up) and TLR7Tg.Bank1 -/- mice. Statistical analysis performed by Mann-Whitney unpaired test. Data representative from 3–5 independent experiments with 5–11 mice per group. Graphs represent mean value with SD.

Supplementary Figure 2 IgA production and bacterial binding in health and lupus. **(A)** Immunofluorescence staining of the IgA expression in the ileum and quantitative analysis of IgA^+^ cells per mm^2^, IgA-FITC (green), Ep-Cam-APC (pink), nuclei-DAPI (blue). **(B)**Frequency of IgA^+^B220^+^ and IgA^+^B220^-^ cells in the PPs from naïve WT and *Bank1-/-* mice by flow cytometry. (c) Frequencies of IgA^+^ cells upon stimulation of naïve B cells (99% purity CD19^+^B220^+^ cells from which 96% were IgD^+^ IgM^+^) from either the PPs (left) or the spleen (right) cultured in the presence with TGFβ, retinoic acid, anti-CD40, and anti-IgM. (D) LEfSe analysis of IgA- fecal bacteria in IMQ-treated mice. Data represents results from 3–5 independent experiments with n=5–11 mice per group. Graphs represent mean value with SD.

Supplementary Figure 3 Gut microbiome composition is modified by *Bank1*. **(A)** Pielou’s eveness, Faith’s PD, and Shannon index for fecal microbiome from naïve WT and Bank1-/- mice. **(B)** Principal component analysis based on the Bray-Curtis analysis of the microbiota composition of single cage naïve and **(C)** IMQ-treated mice. **(D)** ANCOM analysis of fecal microbiome of IMQ-treated WT and *Bank1-/-* mice and **(E)** of TLR7Tg and TLR7Tg. *Bank1-/-* mice. **(F)** Validation of microbiome sequencing by RT-PCR with primers for *P. distasonis* or *B. acidifaciens*.

Supplementary Figure 4 Bank1-deficient microbiota normalization dampens lupus development. **(A)** Assessment of anti-dsDNA total IgG by ELISA in sera. **(B)** Relative frequency of *P. distasonis* in feces. **(C)** Flow cytometry analysis of CD19^+^ B220^+^ and IgA^+^B220^-^ cells in the SILP of naïve littermate mice. **(D)** LDA analysis and LEfse plot of IgA^+^ bacteria sorted and sequenced in IMQ-treated WT littermates and WT single cage mice and **(E)** IMQ-treated *Bank1* ^-/-^ littermate and *Bank1* ^-/-^ single cage mice. **(F)** Correlation of sequenced fecal bacteria and lupus signs and symptoms. Data represent results from 3 independent experiments with n=5–10 mice per group.

Supplementary Figure 5 Oral supplementation with *P. distasonis* ameliorates lupus. **(A)** Correlation between spleen weight and *P.distasonis* abundance in the feces of PBS-gavaged WT mice (Spearman r). **(B)** Proportions of GC B cells in the PPs and **(C)** from the spleen. **(D)** Proportions of IgA^+^ B220^-^ cells in the SILP and **(E)** in the PPs. **(F)** Alpha diversity of gut microbial composition of P. distasonis gavaged mice and control mice that received PBS. **(G)** PCA comparing the beta diversity of the microbiota composition. **(H)** Bar-plots showing the relative frequencies of the taxa composing the gut microbiome. **(I)** Percentages of B220^+^IgA^+^ in PPs and spleen B cells from 20-week-old naïve WT and *Bank1* ^-/-^ mice upon *in vitro* stimulation with P. distasonis. All analyses were done with 4–8 mice per group. Graphs represent mean ± SD.

Supplementary Figure 6 IL-10 production by B cells GALT-resident and in the spleen. **(A)** Gating strategy for the analysis of IL-10-producing B cells. **(B)** Percentages of IL-10^+^ B cells in the spleen of IMQ-treated WT and *Bank1* ^-/-^ mice. **(C)** Median fluorescence intensity of IL-10 expression in B cells from PPs and mLN from IMQ-treated WT and *Bank1* ^-/-^ mice and **(D)** 28–30 weeks old WT, TLR7Tg and TLR7Tg/*Bank1* ^-/-^, **(E)** IMQ-treated SC and LM mice, and **(F)** IMQ-treated and non-treated *P.distasonis* or PBS gavaged mice. **(G)** Percentages of IL-10^+^ B cells in the mLN of IMQ-treated *P.distasonis* or PBS gavaged mice. **(H)** Analysis of the IL-10^+^ production by spleen and mLN B cells from *Bank1* ^-/-^ and WT mice upon *in vitro* stimulation with CpG. **(I)** IL-10 MFI of B cells upon stimulation with CpG. Data represents results from 3–5 different experiments Graphs represent mean ± SD.
