# Supplementary material for: Bank1 deficiency reshapes the gut microbiota of lupus mice towards an anti-inflammatory composition
Source: Front Immunol. 2025 Jul 21;16:1586025. doi: 10.3389/fimmu.2025.1586025 (PMC12318881; doi:10.3389/fimmu.2025.1586025)

Supplementary Figure 1

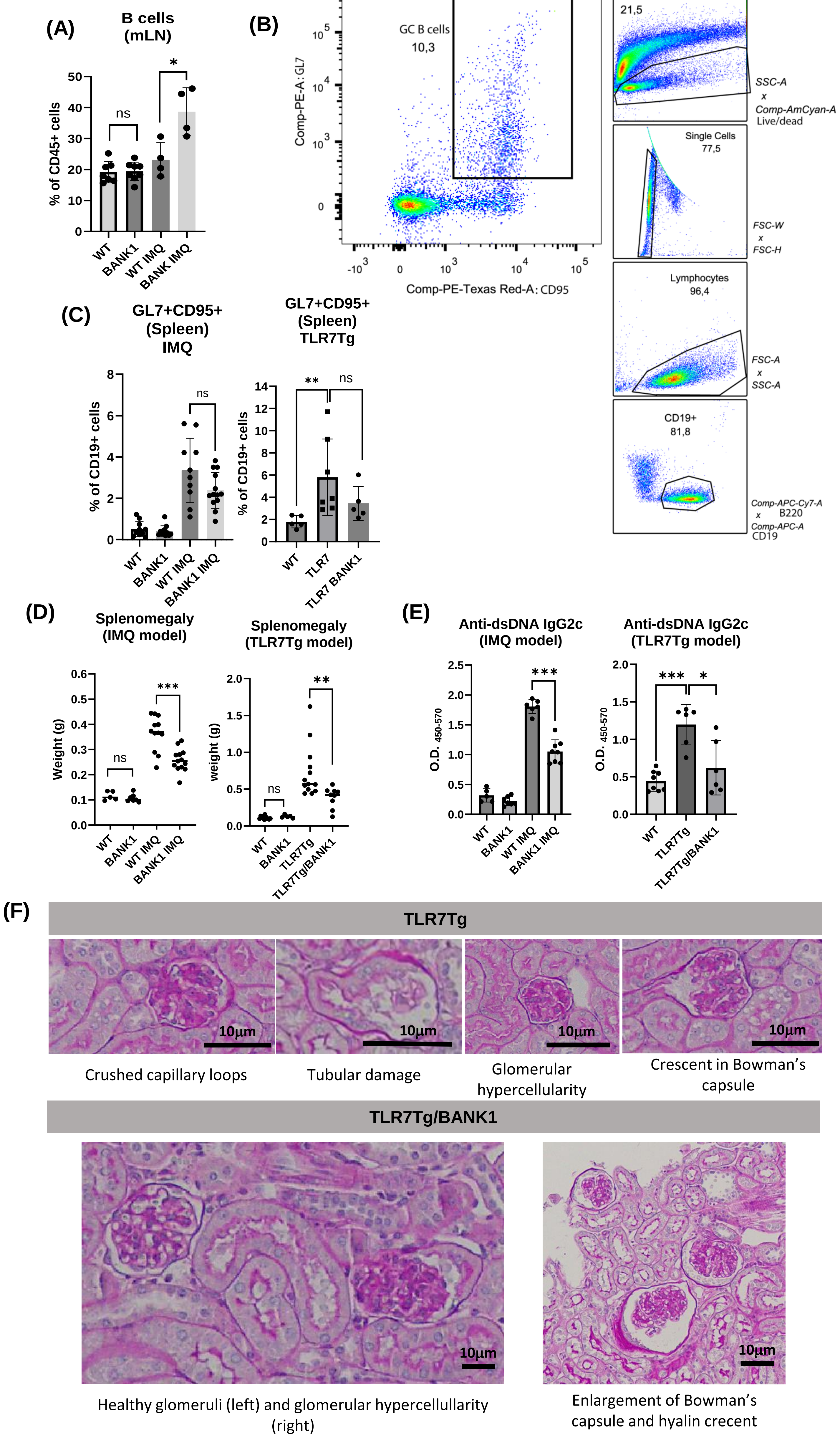

Supplementary Figure 2

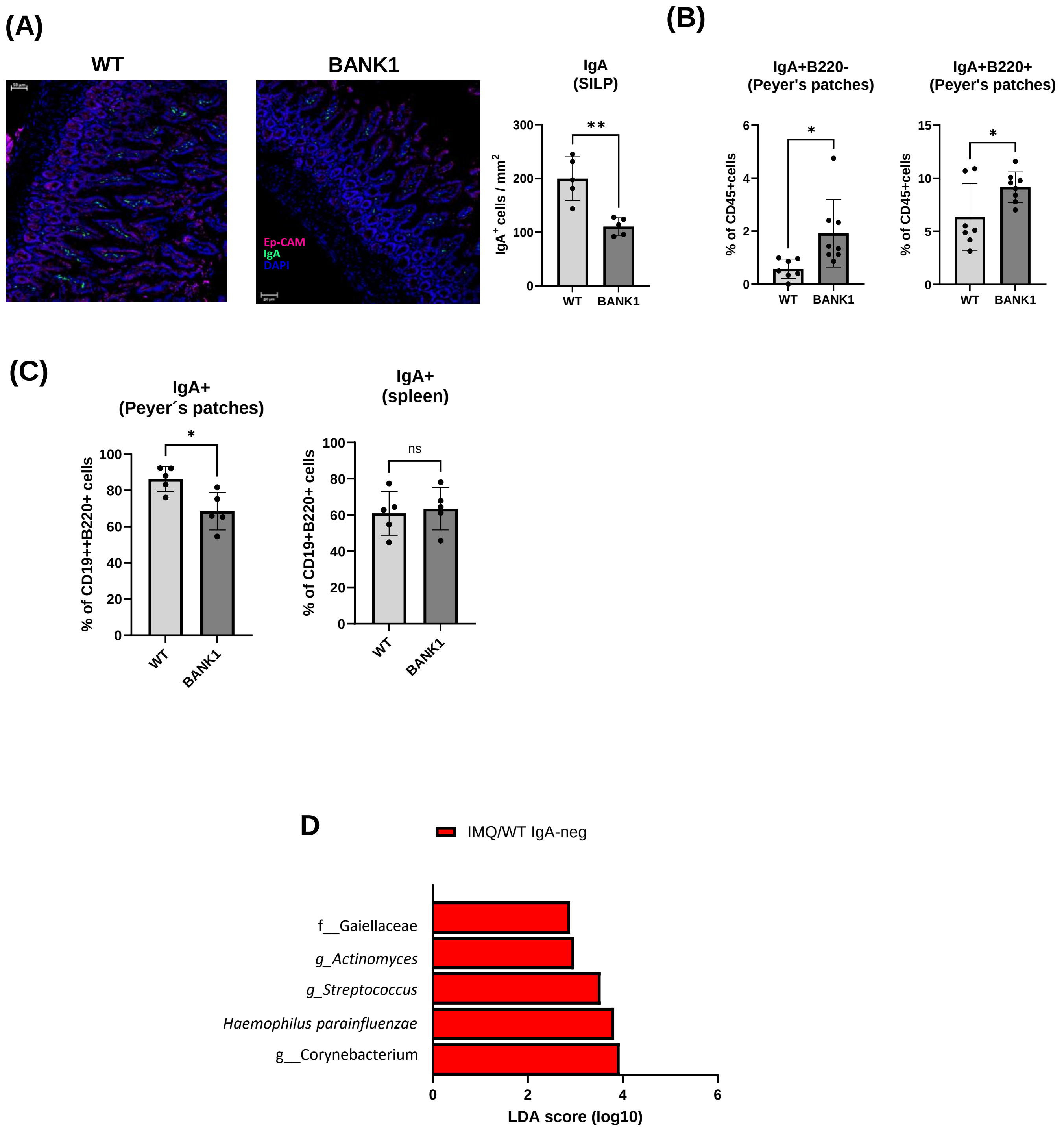

Supplementary Figure 3

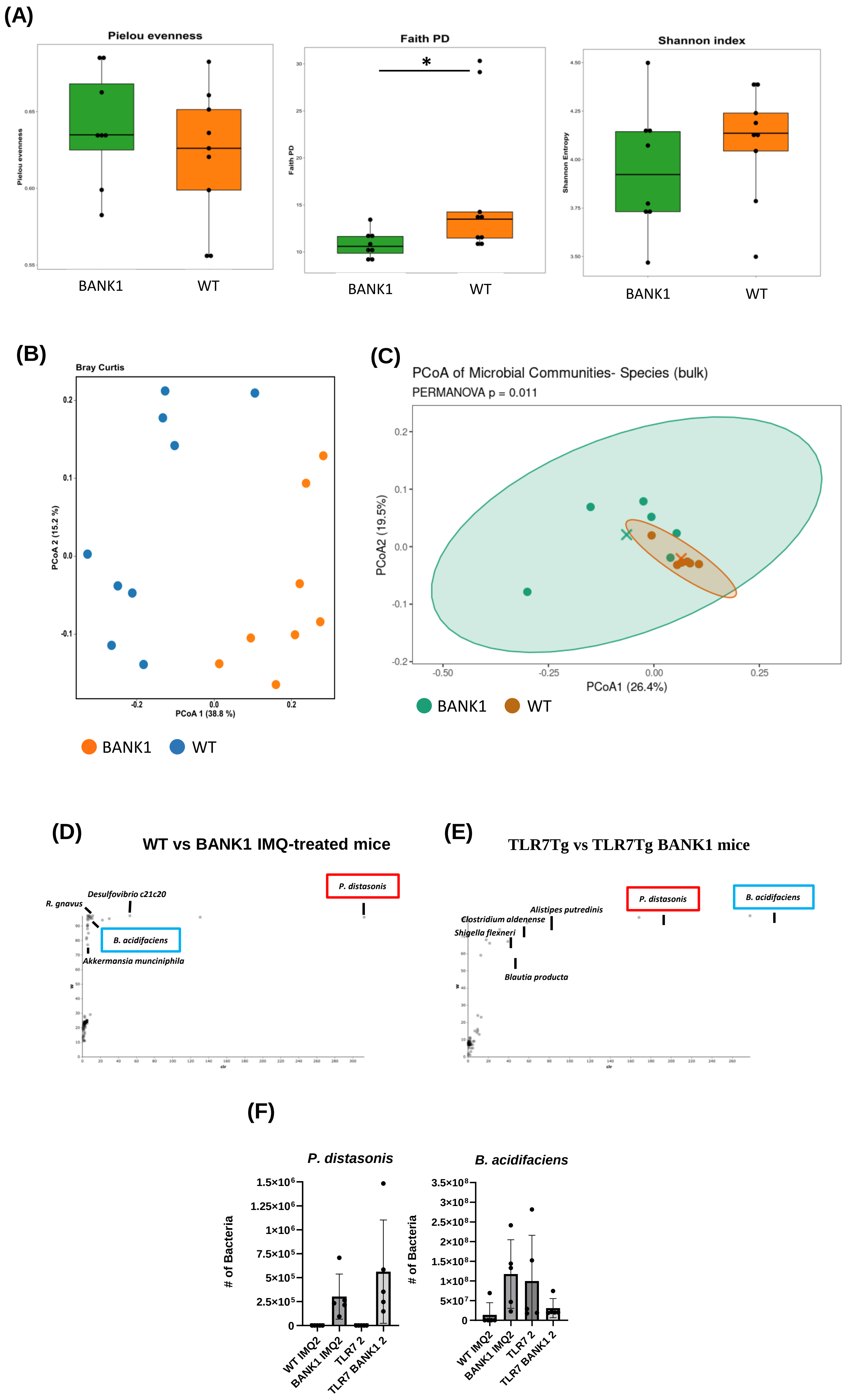

Supplementary Figure 4

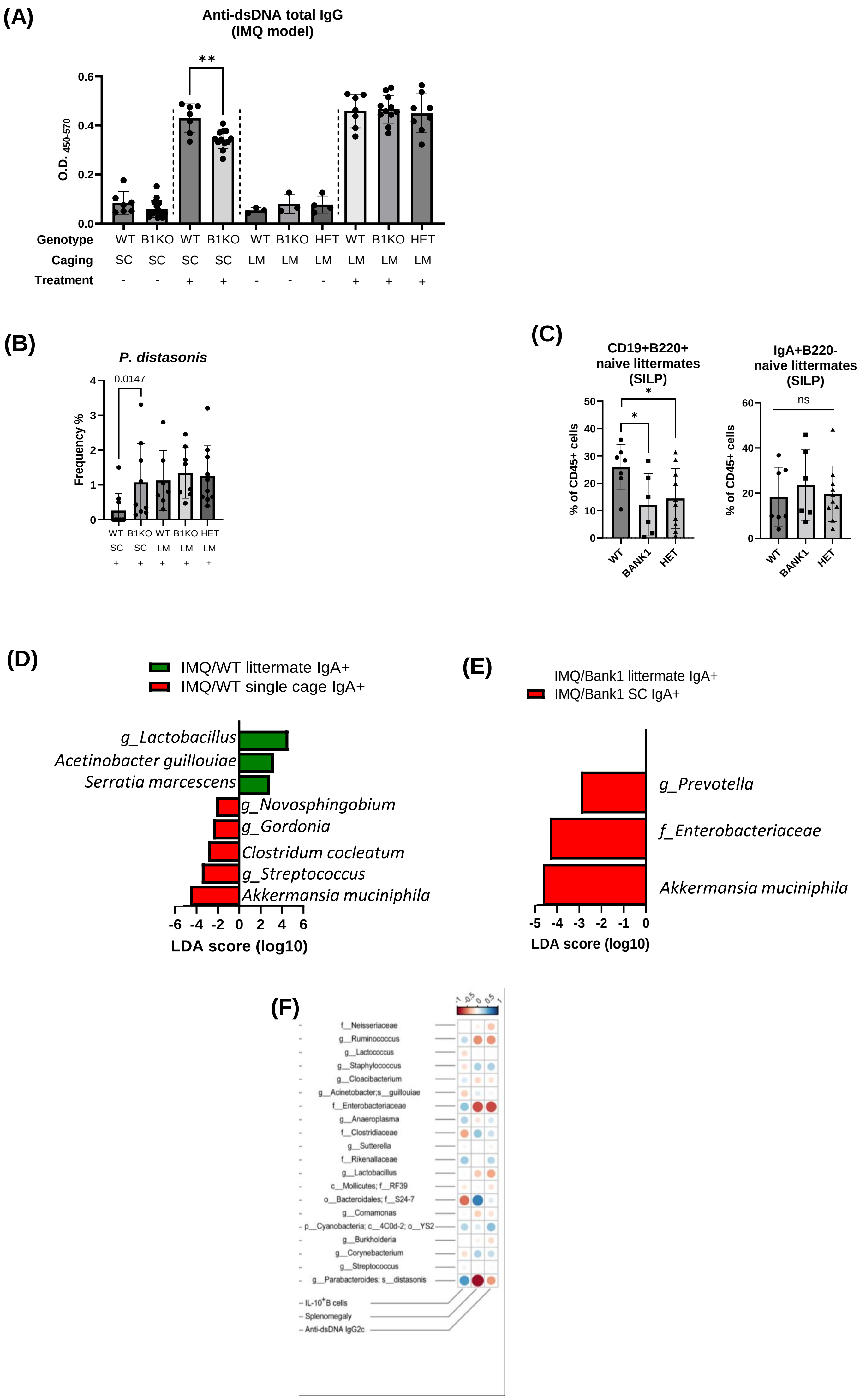

Supplementary Figure 5

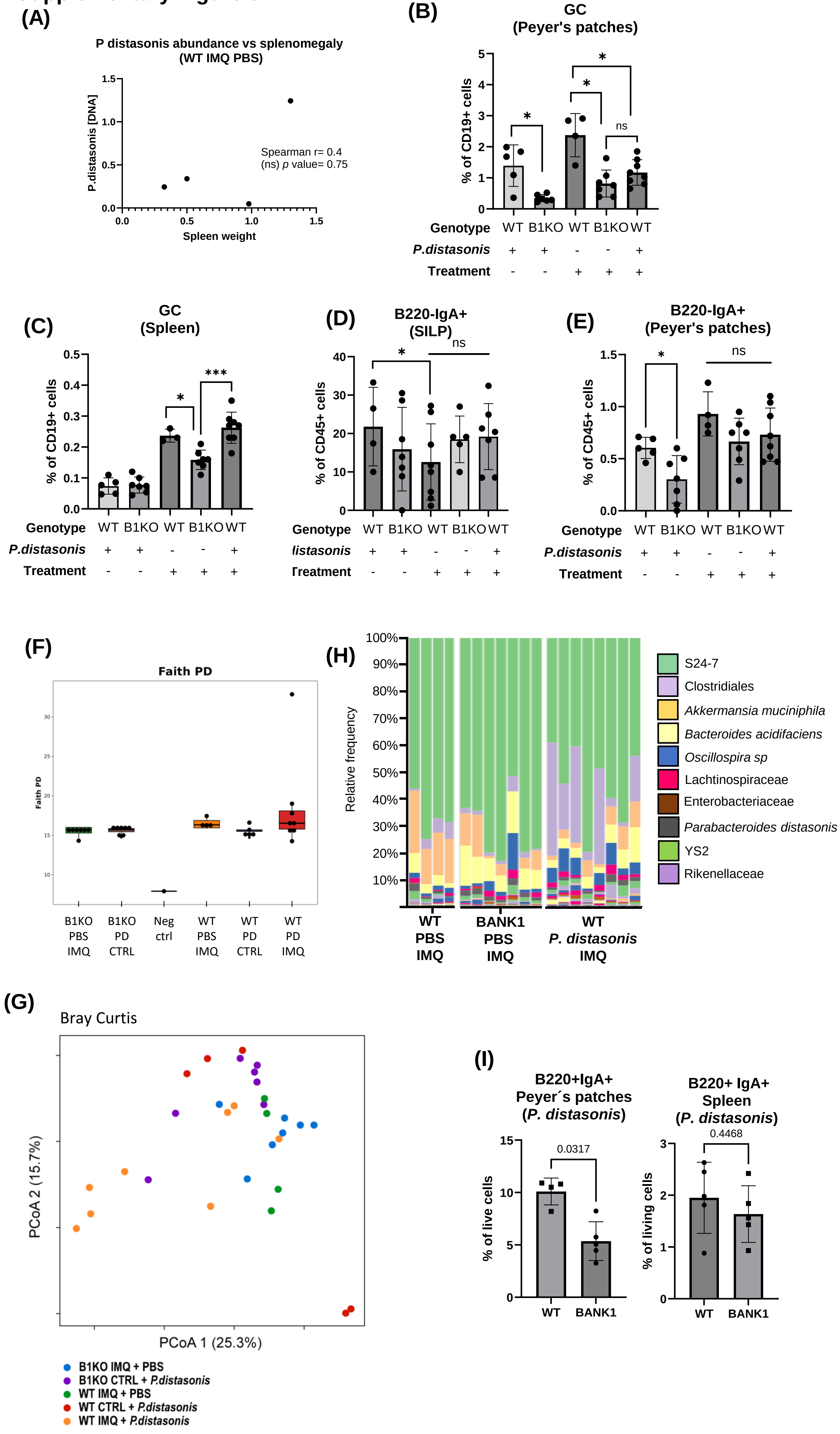

Supplementary Figure 6

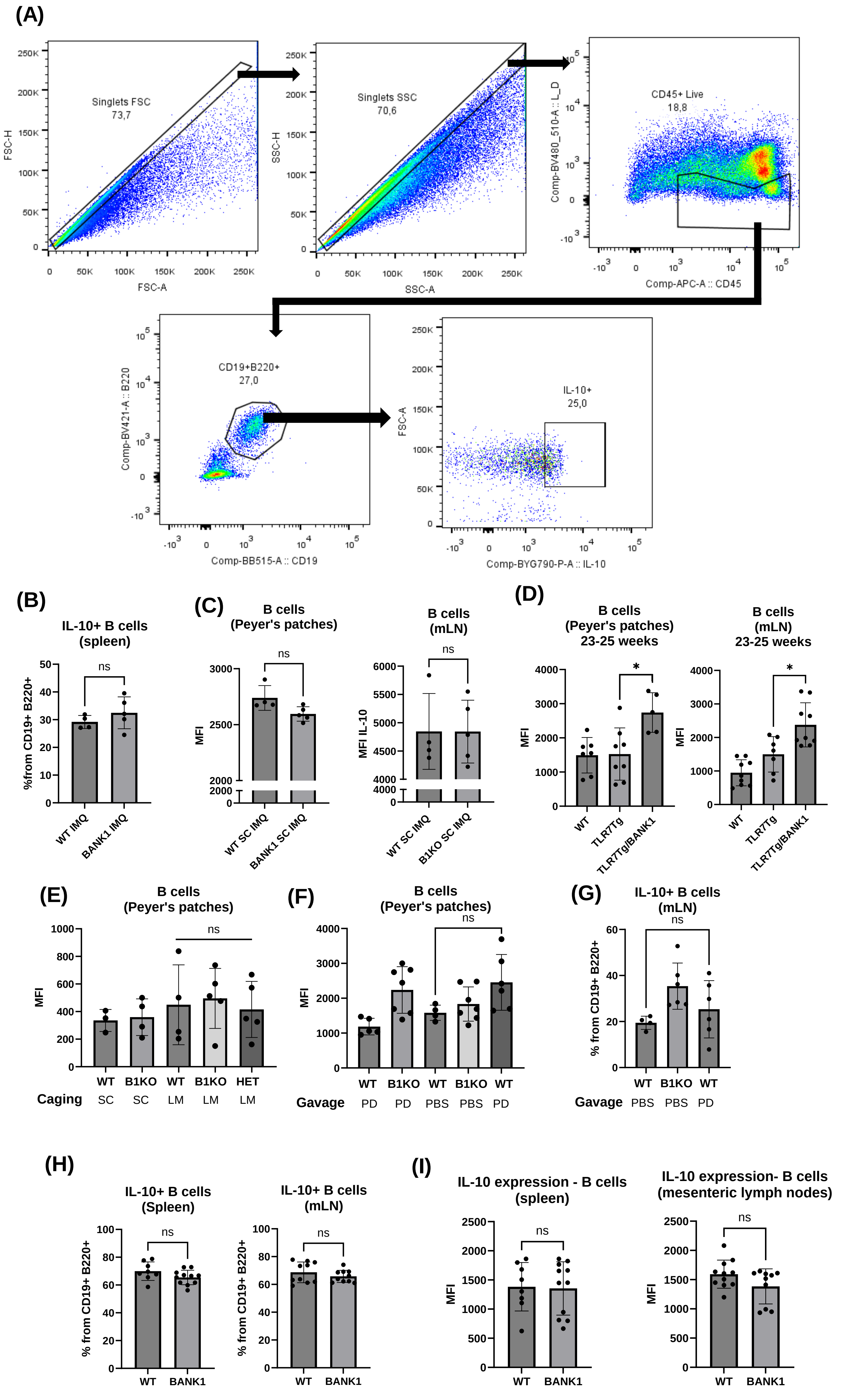

Supplement: Supplementary Figure 1 — Bank1 dampens the gut and systemic inflammation induced by TLR7. (A) Assessment of CD19+B220+ B cells in the mLN of naïve and IMQ-treated WT and Bank1-/- mice. (B) Representation of the gating strategy for GC B cells frequency analysis. (C) GC B cells frequency in the spleen of IMQ-treated WT and Bank1-/- mice and mice from the TLR7Tg model. (D) Spleen weigh of the IMQ model (left) and TLR7Tg model (right). (E) anti-dsDNA IgG2c antibodies in serum in IMQ-treated mice (left) and TLR7Tg lupus model (right). (F) Representative images of the most common renal lesions found in TLR7Tg (up) and TLR7Tg.Bank1 -/- mice. Statistical analysis performed by Mann-Whitney unpaired test. Data representative from 3–5 independent experiments with 5–11 mice per group. Graphs represent mean value with SD. [file DataSheet1.pdf]
